# Anterior insular co-activation patterns associated with stress markers in chronic primary pain

**DOI:** 10.1093/braincomms/fcag121

**Published:** 2026-04-03

**Authors:** Salome Häuselmann, Anna Wyss, Samantha Weber, Nicolas Gninenko, Cristina Concetti, Eliane Müller, Rupert Bruckmaier, Josef Gross, Nina Bischoff, Chantal Berna, Martin grosse Holtforth, Selma Aybek

**Affiliations:** Psychosomatic Medicine, Department of Neurology, Inselspital, Bern University Hospital, University of Bern, 3010 Bern, Switzerland; Graduate School of Cellular and Biomedical Sciences (GCB), University of Bern, 3012 Bern, Switzerland; Translational Imaging Center (TIC), Swiss Institute for Translational and Entrepreneurial Medicine, 3010 Bern, Switzerland; Psychosomatic Medicine, Department of Neurology, Inselspital, Bern University Hospital, University of Bern, 3010 Bern, Switzerland; Translational Imaging Center (TIC), Swiss Institute for Translational and Entrepreneurial Medicine, 3010 Bern, Switzerland; Psychosomatic Medicine, Department of Neurology, Inselspital, Bern University Hospital, University of Bern, 3010 Bern, Switzerland; Department of Adult Psychiatry and Psychotherapy, University Hospital of Psychiatry Zurich, University of Zurich, 8032 Zurich, Switzerland; Faculty of Medicine, University of Zurich, 8032 Zurich, Switzerland; Psychosomatic Medicine, Department of Neurology, Inselspital, Bern University Hospital, University of Bern, 3010 Bern, Switzerland; Department of Neurology, Faculty of Science and Medicine, University of Fribourg, 1700 Fribourg, Switzerland; Department of Neurology, Faculty of Science and Medicine, University of Fribourg, 1700 Fribourg, Switzerland; Psychosomatic Medicine, Department of Neurology, Inselspital, Bern University Hospital, University of Bern, 3010 Bern, Switzerland; Graduate School of Cellular and Biomedical Sciences (GCB), University of Bern, 3012 Bern, Switzerland; Department of Neurology, Faculty of Science and Medicine, University of Fribourg, 1700 Fribourg, Switzerland; Veterinary Physiology, Vetsuisse Faculty, University of Bern, 3012 Bern, Switzerland; Veterinary Physiology, Vetsuisse Faculty, University of Bern, 3012 Bern, Switzerland; Psychosomatic Medicine, Department of Neurology, Inselspital, Bern University Hospital, University of Bern, 3010 Bern, Switzerland; Center for Integrative and Complementary Medicine, Department of Anesthesiology, Lausanne University Hospital, 1011 Lausanne, Switzerland; Psychosomatic Medicine, Department of Neurology, Inselspital, Bern University Hospital, University of Bern, 3010 Bern, Switzerland; Institute of Psychology, University of Bern, 3012 Bern, Switzerland; Department of Neurology, Faculty of Science and Medicine, University of Fribourg, 1700 Fribourg, Switzerland

**Keywords:** chronic primary pain, functional MRI, co-activation patterns, brain dynamics, stress response

## Abstract

Chronic primary pain occurs without an identifiable causal disease and is marked by persistent pain, emotional distress and functional disability. The anterior insular cortex, involved in salience processing and integration of sensory, emotional and cognitive aspects of pain, has been implicated in neural processes linking pain and stress responses. This study investigates whether specific brain state dynamics, using the anterior insula as a seed region, are associated with chronic primary pain and examines their associations with pain- and stress-related measures. Resting-state functional MRI, stress biomarkers (cortisol and alpha-amylase), a pain sensitivity test, as well as subjective measures of stress and pain were collected from patients with chronic primary pain (*N* = 30) and healthy controls (*N* = 30). Co-activation pattern analysis was used to identify brain states with the anterior insula as the seed region and to assess group differences in the temporal characteristics of these brain states. Partial least squares analysis was applied to investigate multivariate associations between specific temporal brain state characteristics and pain- and stress-related measures. Three anterior insula–seeded co-activation patterns (brain states) were identified in healthy controls. In the first co-activation pattern, the anterior insula co-activated with the default mode network; in the second, with the salience-somatomotor network; and in the third, with the visual network. Chronic primary pain patients and healthy controls differed significantly in temporal brain state characteristics, namely in the relative number of entries into co-activation pattern one (*p*_FDR_ = 0.002) and two (*p*_FDR_ = 0.022), and the relative occurrence of co-activation patterns one (*p*_FDR_ = 0.022) and two (*p*_FDR_ = 0.022). Furthermore, in chronic primary pain patients, perceived stress scores and cortisol were significantly associated with these specific temporal brain state characteristics (*P* = 0.002), whereas no associations were found with pain-related measures. Together, these findings suggest that in chronic primary pain, reduced coupling of the anterior insula with the default mode network and increased coupling of the anterior insula with salience-related networks are associated with psychophysiological stress markers. These brain state dynamics may potentially represent a neural correlate of altered stress processing in chronic primary pain.

## Teaser

Häuselmann *et al*. report that chronic primary pain patients show altered anterior insula-based brain-state dynamics. Reduced coupling of the anterior insula with the default mode network and increased coupling with salience-related networks were associated with stress-related measures and may represent neural correlates of altered stress processing in chronic primary pain.

## Graphical Abstract

**Graphical Abstract:**
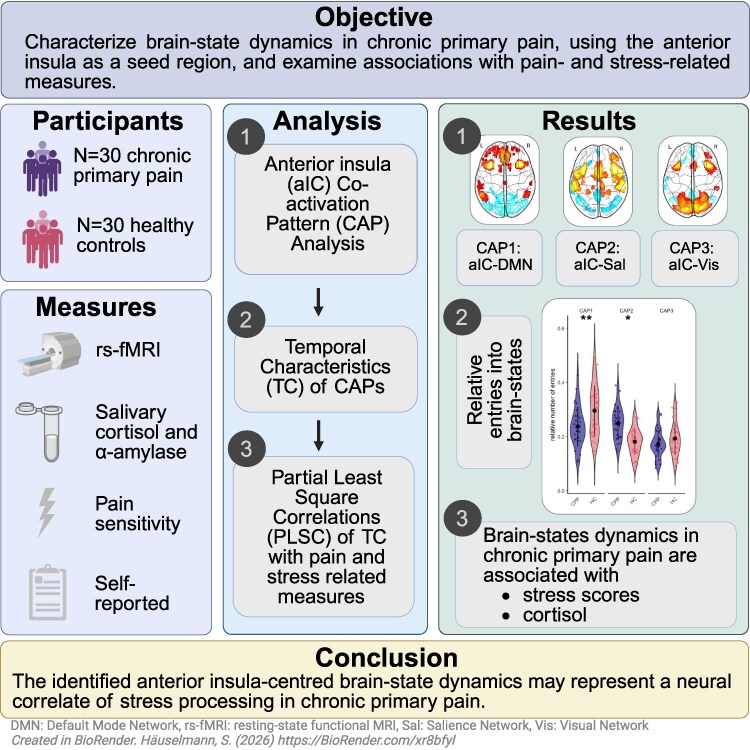

## Introduction

Pain is defined as an unpleasant sensory and emotional experience associated with actual or potential tissue damage or with experiences resembling such damage.^1^ When this experience becomes chronic (i.e. persisting in the absence of ongoing or identifiable tissue damage), pain loses its adaptive role as a warning signal and protective mechanism.^2^ According to ICD-11, chronic primary pain (CPP) conditions—such as chronic widespread pain or chronic primary visceral pain—affect one or more anatomical regions and are associated with significant emotional distress and functional disability.^1^ CPP is a multifactorial condition resulting from an interplay of biological, psychological and social factors.^1,3^ However, the diagnosis of CPP is made regardless of the biological and psychological contributors identified.^4,5^ Despite increasing recognition of its multifactorial aetiology, treatment of CPP remains challenging, with an uncertain prognosis.^6,7^

Notably, both states of acute and chronic stress are considered important contributors to the development and maintenance of chronic pain.^8,9^ Stress-related contributions to pain chronification can be understood within an allostatic load framework, in which early-life vulnerabilities and cumulative stress exposure jointly affect interconnected neural, autonomic and endocrine circuits [e.g. hypothalamic–pituitary–adrenal (HPA) axis] involved in stress and pain regulation.^10,11^ At the level of the brain, these processes are reflected in overlapping networks, particularly within the limbic system, indicating a shared neurobiological basis of both stress and pain processes.^10,12^

Functional magnetic resonance imaging (fMRI) comparing chronic pain populations with healthy controls (HCs) has revealed altered activity and connectivity across multiple brain regions involved in pain processing, emotional regulation and salience detection, such as the insula, thalamus and somatosensory cortex.^9^ A shift in insular activation from the sensory-focused posterior to the affective-focused anterior insular cortex (aIC) has also been observed during the transition from acute to chronic pain, highlighting the aIC’s role in salience detection and integration of sensory, emotional and cognitive aspects of pain.^13-20^ However, such findings are largely based on time-averaged connectivity metrics (i.e. static functional connectivity), which do not capture the inherently dynamic nature of brain network interactions.

Capturing these dynamic and transient changes on a neural level requires approaches that go beyond time-averaged static connectivity or window-based functional connectivity (i.e. classical dynamic functional connectivity). One such method is co-activation pattern (CAP) analysis, a dynamic functional connectivity approach that identifies recurring, transient whole-brain activation patterns (i.e. brain states) in fMRI data at a single-frame level.^21^ CAP analysis thus directly captures the brain’s moment-to-moment reconfiguration of functional networks.^22^ These dynamic patterns are thought to reflect the brain’s functional flexibility and adaptability, and their temporal properties (i.e. temporal characteristics), such as occurrence, duration and transition, offer insight into how neural systems coordinate information over time.^21^ Altered flexibility or stability of brain states have been reported before across neurological and psychiatric disorders,^23-25^ and may also underlie the neural mechanisms associated with CPP. To date, two fMRI studies have applied CAP analyses to chronic pain (sub-)populations.^25,26^ Mawla *et al*.^25^ demonstrated different occurrences of specific CAPs when comparing the resting condition to the sustained pain condition in individuals with fibromyalgia, a condition classified under CPP. Their identified patterns involved co-activation of the salience, dorsal attention and sensorimotor networks, and their temporal dynamics were associated with clinical pain interference and pressure pain sensitivity.^25^ Liu *et al*.^26^ found altered CAP temporal properties, such as time fraction, persistence and transitions, in women with primary dysmenorrhea (i.e. a form of chronic visceral pain), compared to HCs, even during a pain-free phase, suggesting sustained disruptions in brain dynamics. Together, these findings suggest sustained disruptions in the temporal organization of intrinsic brain activity in these chronic pain conditions. While the previous two CAP studies used whole-brain clustering approaches to define CAPs in specific chronic pain subpopulations, the current study employs an aIC-seeded CAP analysis to specifically investigate common transient large-scale network dynamics centred on this key region in CPP patients. Given aIC’s central involvement in salience and stress processing, and the integration of sensory, emotional and cognitive aspects of pain,^13,27^ examining aIC-centred network dynamics may provide deeper insights into stress- and pain-related alterations in intrinsic brain activity in CPP.

By integrating aIC-seeded CAP analysis with clinical measures of pain and stress, we aim to elucidate how altered aIC-seeded brain state dynamics may reflect the interplay between stress and pain at the neural level. We hypothesize that CPP patients will show distinct temporal dynamics of aIC-seeded brain activity compared to HCs, and that these dynamics will be associated with individual differences in pain and stress characteristics.

## Materials and methods

### Participants

The study was conducted at the University Hospital Inselspital Bern, Switzerland. Thirty patients with CPP (ICD-11: MG30.0) and 30 HCs, matched in age and sex, were included between 2023 and 2024. Exclusion criteria were: (i) major neurological comorbidities, (ii) current severe psychiatric condition (acute suicidality, psychosis), (iii) inability to follow study procedures, (iv) history of substance or alcohol abuse, (v) previous brain surgery, (vi) implanted devices, (vii) metal clips in the body and (viii) pregnancy or breastfeeding in women. The study was approved by the Ethics Committee of the Canton Bern (SNCTP000004529, 2020-02283) and was conducted according to the principles of the Declaration of Helsinki. The study protocol was preregistered on ClinicalTrials.gov (NCT05086380). All participants provided written informed consent and were reimbursed for travel expenses.

### Demographic and clinical characteristics

We assessed demographic and clinical variables, including age, sex, smoking status, menstrual cycle phase, menopausal status and medication use. Medications were categorized into psychotropic medications (e.g. benzodiazepines, neuroleptics, antiepileptics, antidepressants, opioids), non-opioid analgesics (e.g. paracetamol, non-steroidal anti-inflammatory drugs), corticosteroids and hormonal contraceptives, considering only those taken on a regular basis. The patient's symptom duration was calculated from the onset of pain to the date of inclusion into the study. Subjective symptom load and acute stress load were assessed using a visual analogue scale (VAS; range: 0–100; 0 = no symptoms/stress; 100 = worst symptoms/stress experienced) on the day of the MRI data acquisition.

#### Self-report questionnaires

Mood was assessed on the day of MRI data acquisition using the State-Trait Anxiety Inventory (STAI), including both the state (STAI-I) and trait (STAI-II) subscales,^28^ as well as the Beck Depression Inventory (BDI).^29^ Sleep quality during the night preceding salivary cortisol collection was evaluated using the total Leeds Sleep Evaluation Questionnaire (LSEQ) score.^30^

Self-reported stress-related questionnaires were administered 1 week (mean 6.4 ± 5.2 days) before MRI data acquisition. Early life stress was assessed with the 28-item Childhood Trauma Questionnaire (CTQ),^31^ which captures five subtypes of childhood adversity: emotional abuse, physical abuse, sexual abuse, emotional neglect and physical neglect. Acute levels of perceived stress were measured with the 10-item Perceived Stress Scale (PSS),^32^ which measures how frequently participants experienced specific thoughts and feelings related to stress load over the past month.

Self-reported pain-related questionnaires were administered on the day of MRI data acquisition: The Widespread Pain Index (WPI) assessed the number of painful body regions over the last week.^33^ The Brief Pain Inventory (BPI)^34^ was used to collect the current pain intensity, pain severity (four items addressing the worst, least and average pain experienced over the past 24 h, as well as current pain) and pain interference (seven items, addressing how much the pain interferes with daily life).

#### Pain provocation test

Pain sensitivity was assessed using a standardized and validated pain provocation test with peg algometry (Algopeg)^35,36^ after the MRI data acquisition. The standardized polypropylene and nickel pegs (78 × 10 mm^2^) were calibrated to exert a clamping force of 10 N at a 5 mm extension. The test was applied to both middle fingers (i.e. on the nail bed without contacting the nail fold) and earlobes (i.e. on the central soft tissue without touching the ear cartilage) of each participant. After 10 s of applied clamping force, participants were asked to rate their pain intensity on a numeric rating scale ranging from 0 (=no pain) to 10 (=worst pain imaginable). If a participant was unable to endure the full 10-s duration, the test was prematurely terminated with a rating of 10. Pressure applied to the middle fingers is typically perceived by patients as being beneath or slightly above the pain threshold, capturing the transition from non-painful to painful sensation. In contrast, pressure to the earlobe was generally rated as clearly above the pain threshold, serving as a measure of pain endurance and tolerance.^35^ For further analysis, we calculated the overall mean by combining data from both the fingers and earlobes.

#### Salivary cortisol and alpha-amylase

To assess objective stress biomarkers, saliva samples were collected for the analysis of cortisol and alpha-amylase. The salivary cortisol awakening response (CAR) is characterized by a rapid increase in cortisol secretion within 30–45 min after awakening and is considered a sensitive marker of HPA axis activity, reflecting endocrine stress system function.^37-39^ Whereas salivary alpha-amylase, a digestive enzyme secreted by the salivary glands, serves as an indicator of stress reactivity and reflects rapid autonomic nervous system (ANS) activity, particularly the sympathetic-adrenal-medullary response.^40^ The samples were obtained using cotton swabs (Salivette Collection Devices, Sarstedt), with participants holding the swab in their mouth for ∼1 min per sample. Samples for the cortisol analysis were collected 1 day (mean 1.3 ± 1.5 days) before the MRI data acquisition, following the guidelines of Stalder *et al*.,^39^ including refraining from strenuous physical activities, heavy meals, fruits or fruit juices, coffee, carbonated soft drinks, chewing gum and smoking. Participants were instructed to collect five saliva samples over the course of a single day; samples were taken immediately upon awakening, and subsequently at 15, 30, 45 and 60 min post-awakening. Salivary samples for alpha-amylase analysis were collected immediately before and after the MRI session. All saliva samples were centrifuged at room temperature for 10 min at 3500 rpm and subsequently stored at −20 °C until analysis. Salivary cortisol and alpha-amylase concentrations were determined using commercially available saliva-specific enzyme immunoassays (Salimetrics, High Sensitivity Salivary Cortisol Enzyme Immunoassay kit, 1-3002; Salimetrics, Salivary Alpha-Amylase Kinetic Enzyme Assay Kit, 1-1902), following the manufacturer’s protocols. As a measure for the CAR, we calculated the area under the curve with respect to the increase (AUC_I_) based on the five cortisol samples for each participant.^39,41^ Alpha-amylase concentrations were measured in pre- and post-MRI samples for each participant, and the mean of these two values was computed per individual.

### MRI data acquisition and preprocessing

Structural and resting-state MRI data were acquired using a 3 tesla scanner (Magnetom Prisma, Siemens, Germany). The structural scan consisted of a sagittal-oriented T1-weighted 3D-magnetization-prepared rapid acquisition gradient echo (MPRAGE) sequence with 1 mm isotropic voxel size [repetition time (TR) = 2330 ms, echo time (TE) = 3.03 ms, inversion time (TI) = 1100 ms, flip angle (FA) = 8°, matrix = 256 × 256 × 176]. The resting-state functional scan consisted of a whole-brain, interleaved, multislice echo-planar imaging sequence with an isotropic voxel size of 2.2 mm (TR = 1300 ms, TE = 37 ms, FA = 52°, matrix size = 104 × 104 × 60), yielding a total of 300 functional volumes. During the fMRI acquisition, participants were instructed to fixate on a white cross displayed on a black background and to avoid engaging in specific thoughts or falling asleep. MRI data were preprocessed using SPM12 (Wellcome Centre for Human Neuroimaging, UCL, London) in MATLAB (R2023a, MathWorks Inc., Natick, USA). Functional volumes were initially realigned and unwarped (unwarping step omitted for two subjects due to corrupted files), and subsequently co-registered to the corresponding structural T1-weighted image. Single-voxel time series were detrended and corrected for nuisance contributions by regressing out the average white matter and cerebrospinal fluid signals, the six motion parameters (three translations and three rotations), the global signal and low-frequency trends (constant, linear and quadratic). A high-pass filter with a cutoff frequency of 0.01 Hz was then applied to remove low-frequency drifts. Finally, functional volumes were warped into MNI standard space and spatially smoothed using a Gaussian kernel with a full width at half maximum (FWHM) of 5 mm. Functional images were assessed for excessive head motion using the framewise displacement (FD) criterion,^42^ with a threshold of FD > 0.5 mm. No subject exceeded the exclusion threshold of having more than 40% of volumes with FD > 0.5 mm.

### Resting-state functional dynamics

#### Co-activation patterns

**Figure 1:**
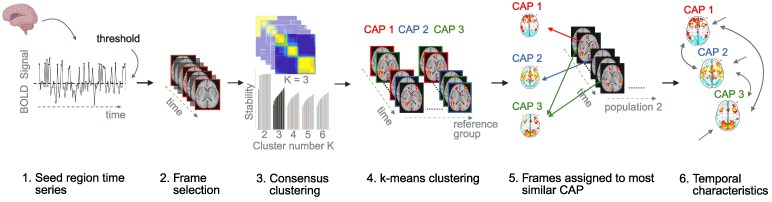
**Workflow of CAP analysis.** (i) The average BOLD signal time series from the seed region is extracted. (ii) High-amplitude frames (i.e. activations) of the seed signal are selected based on a predefined threshold. (iii) The optimal number of clusters (*K*) is determined using consensus clustering on the selected frames from the reference group (e.g. HCs). (iv) The selected frames are clustered into K distinct states (CAPs) using the k-means algorithm, based on the optimal K determined through consensus clustering (step 3). (v) A frame from a second population (e.g. CPP patients) is assigned to a CAP if its spatial correlation with that CAP exceeds the 5th percentile of the corresponding distribution from the reference group. (vi) Temporal characteristics of the identified CAPs (e.g. occurrence rate, entries, duration) are calculated and compared between groups. Abbreviations: BOLD signal = blood-oxygen-level-dependent signal, CAP = co-activation pattern, CPP = chronic primary pain, HC = healthy control. Created in BioRender. Häuselmann, S. (2026) https://BioRender.com/5zp80bo.

CAP analysis allows the identification of transient co-(de)activation patterns (i.e. brain states) that recur throughout a resting-state time series. This method captures both the spatial and temporal information of dynamic brain activity.^21,22,43^ We applied a seed-based CAP approach rather than a whole-brain CAP approach to obtain an interpretable state space focused on the region of interest. To generate the CAPs, we used the TbCAPs toolbox (https://github.com/MIPLabCH/TbCAPs), as detailed in Bolton *et al*.^21^ Visualizations of the CAPs were generated using the AtlasReader package (https://github.com/miykael/atlasreader) in Python (version 3.12.7). The aIC was selected as the seed region of interest, given its role in integrating sensory, emotional and cognitive aspects of pain.^13^ A bilateral aIC mask was created in SPM12 based on the procedure by Zhang *et al*.,^44^ using the Human Brainnetome Atlas (https://atlas.brainnetome.org). From the preprocessed fMRI data, the seed time series was extracted, normalized using *z*-scoring and thresholded at 0.84 SD to identify time points corresponding to high-amplitude events (i.e. activations) in the aIC blood-oxygen-level-dependent (BOLD) signal.^21^ Frames exceeding an FD threshold of 0.5 mm, along with the adjacent −1 and +1 frames, were scrubbed from the data. An additional principal component analysis (PCA) step was applied to the retained frames to reduce the high dimensionality of the data and the resulting computational load (this step is described in more detail in Weber *et al*.^24^). To determine the optimal number of clusters *K*, we performed consensus clustering on the retained frames from HCs (reference population), as recommended when differences in activity patterns are expected in the clinical population.^21,45^ The PCA-projected data served as input for the consensus clustering procedure. The optimal cluster size was identified based on consensus clustering metrics, including the inspection of the proportion of ambiguously clustered pairs (PAC) and the visual inspection of consensus matrices^46^ (see the Supplementary Figs 1 and 2). The dimensionality-reduced fMRI volumes were then clustered into three distinct states (CAPs) using the k-means algorithm (50 repetitions), as this cluster number was identified as the most stable. The individual CAPs were reconstructed by reversing the dimensionality reduction. The resulting CAPs were then spatially *z*-scored, reflecting distinct aIC CAPs with both positive and negative contributions. Significant voxels within each CAP were quantified by computing their spatial overlap with networks from the Yeo atlas.^47^ Each retained fMRI frame from CPP patients was matched to the most similar CAP based on spatial correlation. The highest spatial correlation between a given CPP frame and the CAPs was identified and then compared to the distribution of spatial correlations from HC frames associated with that CAP.^21^ If the CPP frame’s correlation exceeded the 5th percentile of this HC distribution, it was assigned to the corresponding CAP.^21^ The whole CAP analysis workflow is illustrated in Fig. 1. The analyses were repeated with the second most stable cluster number (Supplementary Fig. 3). Additionally, for comparison, a static functional connectivity analysis with the aIC as seed region was performed in the Supplementary Material E (Supplementary Table 5, Supplementary Fig. 11).

#### Temporal characteristics of CAPs

To characterize the temporal properties of the identified CAPs, we computed three metrics. (i) Average duration, reflecting how long a brain state remains stable once it is entered, calculated as the mean number of consecutive volumes assigned to a CAP, multiplied by the TR. (ii) Relative number of entries indicating how often a subject transitions into a specific CAP, calculated as the number of transitions into that CAP normalized by the number of retained frames. (iii) Relative occurrence, representing the overall proportion of volumes assigned to a given CAP, calculated as the number of volumes assigned to that CAP normalized by the number of retained frames.

Reduced temporal characteristics of CAPs may indicate less frequent co-activation with the seed, reflecting decreased coupling, whereas increased temporal characteristics may suggest enhanced coupling.^22,48,49^ In contrast, reduced temporal characteristics of co-deactivation patterns may indicate weaker anti-correlation, reflecting transiently increased coupling, whereas increased temporal characteristics may suggest stronger transient anti-correlations, reflecting reduced coupling.^22,48,49^

### Statistical analysis

Statistical analyses were performed in MATLAB (R2023a, MathWorks Inc., Natick, MA, USA) and RStudio (2024.12.1, Posit PBC, Boston, MA, USA).

To analyse the demographic and clinical data, as well as the number of retained frames, we used Pearson’s *χ*^2^ test, Fisher's exact test, Welch’s two-sample *t*-test, or the Wilcoxon rank-sum test, depending on the type of data, distributional assumptions (assessed with the Shapiro–Wilk test) and homogeneity of variances (assessed with the *F*-test).

To examine group differences in the temporal characteristics of the CAPs, linear mixed-effects models were fitted with a group-by-state interaction term as the main fixed effect and subject ID included as a random intercept. Age, sex, psychotropic medication (binary), combined BDI/STAI-II sum score and the number of selected frames were included as covariates for the model. These covariates were included to mitigate potential confounding by comorbid affective symptoms and medication use in group comparisons of CAP temporal characteristics. As a robustness check, we fit a minimally adjusted model (age, sex, selected frames) and an extended sensitivity model, additionally adjusting for non-opioid analgesic use (see Supplementary Table 3). *Post hoc* pairwise comparisons between groups were performed for each CAP state using linear contrasts. *P*-values from the three linear models were adjusted for multiple comparisons across models using the false discovery rate (FDR) method, and results were considered significant at *q*_FDR_ < 0.05. Cohen’s *d* was calculated as a measure of effect size.

To identify multivariate correlation patterns between CAPs’ temporal characteristics and stress- and pain-related measures, we conducted a Partial Least Squares Correlation (PLSC) analysis using the publicly available MATLAB-based PLS toolbox (https://github.com/FND-ResearchGroup/myPLS_SL).^50^ Three CPP patients were excluded from the analysis involving stress-related measures due to deviations from the saliva sampling protocol, including missing samples and/or delays (Δ*t* > 5 min). This method identifies latent variables that optimally capture the covariance between two multivariate datasets by computing weighted linear combinations of CAPs’ temporal characteristics (relative entries and occurrences for each CAP showing group differences) and stress- or pain-related measures (e.g. perceived stress score, BPI severity score).^51,52^ The weights (saliences) reflect the contribution of each variable to these multivariate associations. The statistical significance of the associations was assessed via permutation testing (2000 permutations), and the robustness of the saliences was evaluated through bootstrapping (500 resamples with replacement). We interpret the saliences (i.e. PLS correlation weights) as reflecting the contribution of each variable to the multivariate association between CAPs’ temporal characteristics and stress- or pain-related measures. Since the data were standardized, the saliences can be interpreted similarly to correlation coefficients. Additionally, to examine direct associations between CAPs’ temporal characteristics and individual pain-related measures, univariate correlation analyses were performed. For both the PLSC and the univariate correlation analysis, residuals were used for those variables where covariates of no interest had been regressed out. Specifically, CAPs’ temporal characteristics were adjusted for age, sex, psychotropic medication (binary), combined BDI/STAI-II sum score and the number of selected frames as described previously. Alpha-amylase and AUC_I_ CAR were adjusted for age, sex, contraceptive use, menstrual cycle phase and smoking. Peg algometry scores were adjusted for age, sex, psychotropic medication and non-opioid analgesics use.

## Results

### Demographics and clinical characteristics

Table 1 summarizes the demographic and clinical characteristics of the 30 patients with CPP (ICD-11: MG30.0) and the 30 HCs.

**Table 1:** Demographic and clinical data of patients with CPP and HCs

	CPP (*N* = 30)	HC (*N* = 30)	Statistics^a^
Age (mean, SD)	41.3 (11.6)	40.9 (13.8)	ns
Sex, females/males	24/6	24/6	ns
Smoker, yes/no	9/21	1/29	χ^2^(1) = 7.68, *P* < 0.01
Menopause, yes/no	4/20	3/21	ns
Menstrual cycle	Anovulation 6	Anovulation 5	ns
	Follicular 4	Follicular 1	
	Luteal 12	Luteal 11	
	Menstruation 2	Menstruation 3	
	Ovulation 0	Ovulation 4	
**Medication** (yes/no)			
Psychotropic medication	16/14	1/29	χ^2^(1) = 18.47, *P* < 0.001
Non-opioid analgesics	6/24	0/30	χ^2^(1) = 6.67, *P* < 0.01
Corticosteroids^b^	2/28	0/30	ns
Hormonal contraception	6/18	8/16	ns
**Psychological scales** (mean, SD)			
BDI, score	18.5 (9.8)	4.5 (3.6)	*W* = 861, *P* < 0.001
STAI-I, score	51.1 (11.8)	31.8 (7.0)	*t*(46.91) = 7.71, *P* < 0.001
STAI-II, score	53.3 (11.2)	33.9 (7.8)	*t*(51.70) = 7.78, *P* < 0.001
LSEQ total, score	45.2 (13.9)	52.5 (9.5)	*t*(51.16) = −2.35, *P* < 0.05
**Stress-related measures** (mean, SD)			
CTQ total, score	50.6 (21.0)	35.2 (10.9)	*W* = 677, *P* < 0.001
Acute stress load, score	49.3 (27.4)	19.1 (19.0)	*W* = 733.5, *P* < 0.001
PSS, score	24.5 (8.1)	12.7 (4.5)	*W* = 797.5, *P* < 0.001
alpha-amylase [U/ml]	168.0 (90.4)	197.0 (179.0)	*W* = 490, ns; ^c^*β* = 38.18, SE = 41.31, *t*(50) = 0.92, *P* = 0.360
CAR AUC_I_	105.0 (104.0)	47.7 (93.2)	*W* = 540, *P* < 0.05; ^c^*β* = −50.83, SE = 31.03, *t*(47) = −1.64, *P* = 0.108
**Pain-related measures** (mean, SD)			
Symptom duration, years	13.6 (12.0)	NA	NA
Subjective symptom load, score	50.1 (22.4)	NA	NA
WPI total, score	8.7 (3.9)	NA	NA
Pain intensity (current), score	4.5 (2.0)	NA	NA
BPI severity, score	4.6 (1.7)	NA	NA
BPI interference, score	5.6 (2.0)	NA	NA
Peg algometry—mean, score	4.79 (2.04)	3.06 (1.29)	*t*(49.10) = 3.93, *P* < 0.001 ^d^*β* = −1.13, SE = 0.54, *t*(54) = −2.09, *P* = 0.041

^a^Pearson’s *χ*^2^ test, Fisher's exact test, Welch two-sample *t*-test, Wilcoxon rank-sum test.

^b^Corticosteroid treatment was limited to topical administration (nasal spray) and was not administered on the day of saliva sampling.

^c^Group differences in alpha-amylase and CAR AUC_I_ were analysed using a linear model, adjusted for age, sex, hormonal contraception, menstrual cycle and smoking. Three patients were excluded from this CAR AUCi analysis due to deviation from the saliva protocol.

^d^Group differences in peg algometry were analysed using a linear model, adjusted for age, sex, psychotropic medication and non-opioid analgesics.

AUCi = area under the curve with respect to increase, BDI = Beck’s Depression Inventory, BPI = Brief Pain Inventory, CAR = cortisol awakening response, CPP = chronic primary pain, CTQ = Childhood Trauma Questionnaire, HC = healthy controls, LSEQ = Leeds Sleep Evaluation Questionnaire, ns = not significant, NA = not applicable, SE = standard error, SD = standard deviation, STAI-I = State-Trait Anxiety Inventory, State version, STAI-II = State-Trait Anxiety Inventory, Trait version, WPI = Widespread Pain Index.

CPP patients had a mean symptom duration of 13.6 years (SD = 12.0). Compared to HCs, CPP patients showed significantly higher scores on stress-related measures, including the CTQ (*W* = 677, *P* < 0.001), acute stress load ratings (*W* = 733.5, *P* < 0.001), PSS scores (*W* = 797.5, *P* < 0.001) and cortisol AUC_I_ of the CAR (*W* = 540, *P* = 0.032). In contrast, mean alpha-amylase concentrations did not differ significantly between groups (*W* = 490, *P* = 0.559). Regarding pain-related measures, CPP patients exhibited significantly higher pain sensitivity, as assessed by mean peg algometry (*t*(49.10) = 3.93, *P* < 0.001), compared to HCs.

### Identified aIC-based CAPs in HC

**Figure 2:**
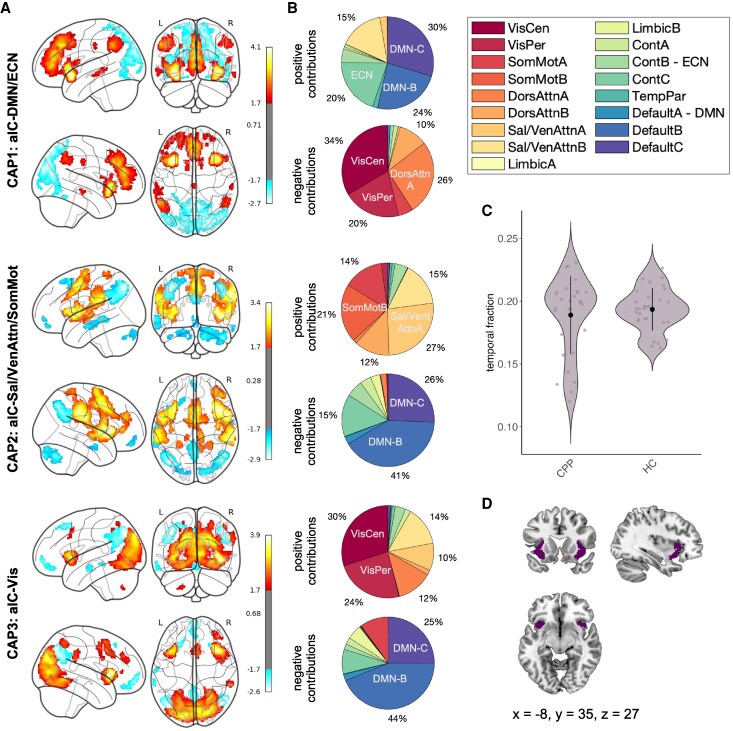
**CAP maps based on aIC seed activation.** (**A**) Three CAPs were detected using HCs as the reference population (*N* = 30). CAPs were *z*-scored, and only the top 5% most positive and the top 5% most negative contributions are shown (*z* > ±1.65), with red indicating co-activation and blue indicating co-deactivation. The locations are shown using the standard coordinates in the MNI space. (**B**) Pie charts show the percentage of 15% top positive and 15% top negative contributions within the 17 resting-state networks as defined by the Yeo atlas (*z* > ±1.04). The seed voxels have been excluded. (**C**) Violin plots show the distribution of the fraction of retained fMRI volumes with anterior insula activation thresholded at 0.84 SD in patients with CPP (*N* = 30) and HC (*N* = 30). A Wilcoxon rank-sum test indicated no significant difference in the fraction of retained frames between groups (*W* = 477, *P* = 0.694). (**D**) Mask for the anterior insular cortex seed region in MNI space. Abbreviations: aIC = anterior insular cortex, Cont = executive control, CPP = chronic primary pain, Default = default mode, DorsAttn = dorsal attention, HC = healthy control, MNI = Montreal Neurological Institute, SomMot = somatomotor, TempPar = temporoparietal, VisCen = central visual, VisPer = peripheral visual, Sal/VenAttn = salience/ventral attention, SD = standard deviation.

The number of retained fMRI volumes, exceeding the aIC threshold (*Z* > 0.84), did not differ significantly between groups (mean_CPP_ = 56.7, SD_CPP_ = 9.35, mean_HC_ = 58.0, SD_HC_ = 5.06, *W* = 477, *P* = 0.694; Fig. 2C, Supplementary Table 1) and was therefore used for the normalization of the temporal characteristics.

Three aIC-seeded CAPs were identified and defined using HCs (*n* = 30) as the reference population (Fig. 2A). In CAP1, the aIC was co-activated with the default mode network (DMN) and executive control network (ECN) and co-deactivated with the visual and dorsal attention network (Fig. 2B, Supplementary Table 2). In CAP2, the aIC was co-activated with the salience/ventral attention and somatomotor network and co-deactivated with the DMN (Fig. 2B, Supplementary Table 2). In CAP3, the aIC was co-activated with the visual network and co-deactivated with the DMN (Fig. 2B, Supplementary Table 2).

As a control condition, we conducted a CAP analysis using the whole insula. This analysis did not yield a clear optimal cluster number, as indicated by a high number of ambiguous cluster pairs (see Supplementary Figs 4 and 5), likely reflecting the heterogeneous functional roles of the insula.

### CPP is characterized by altered CAPs’ temporal dynamics

**Figure 3:**
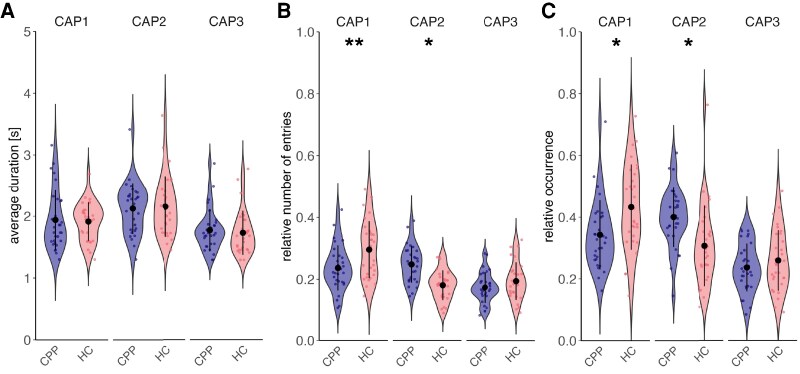
**Temporal characteristics of CAP1, CAP2 and CAP3 in CPP patients and HCs.** (**A**) Average duration, (**B**) relative entries, (**C**) relative occurrence. Violin plots show the distribution of raw data from 30 CPP patients and 30 HCs, with mean and SD indicated. Linear mixed-effects models were fitted, controlling for covariates of no interest. *Post hoc* pairwise comparisons between groups were conducted, and *P*-values were corrected for multiple comparisons using FDR. The relative number of entries and the relative occurrence of CAP1 were lower in CPP patients than in HCs (estimate = 0.07, SE = 0.02, *p*_FDR_ = 0.002; estimate = 0.09, SE = 0.04, *p*_FDR_ = 0.022), whereas CAP2 showed higher relative entries and relative occurrence in CPP patients compared with HCs (estimate = −0.05, SE = 0.02, *p*_FDR_ = 0.022; estimate = −0.09, SE = 0.04, *p*_FDR_ = 0.022). *P < 0.05, **P < 0.01. Abbreviations: CAP = co-activation pattern, CPP = chronic primary pain, FDR = false discovery rate, HC = healthy control, SD = standard deviation, SE = standard error.

To compare the temporal characteristics of each CAP between 30 CPP patients and 30 HCs, we analysed average duration, relative number of entries and relative occurrence. CPP patients entered CAP1 relatively less often than HCs (estimate = 0.07, SE = 0.02, *p*_FDR_ = 0.002, Cohen’s *d* = 1.13), whereas they entered CAP2 relatively more often than HCs (estimate = −0.05, SE = 0.02, *p*_FDR_ = 0.022, Cohen’s *d* = −0.82) (Fig. 3B). Additionally, the relative occurrence of CAP1 was lower in CPP patients compared to HCs (estimate = 0.09, SE = 0.04, *p*_FDR_ = 0.022, Cohen’s *d* = 0.82), whereas CAP2 occurred more frequently in CPP patients than HCs (estimate = −0.09, SE = 0.04, *p*_FDR_ = 0.022, Cohen’s *d* = −0.80) (Fig. 3C). No significant group differences were observed in the average duration of any CAP (Fig. 3A, and Supplementary Table 3). Sensitivity analyses confirmed that the findings on CAP temporal characteristics were robust to covariate adjustments (Supplementary Table 3).

### CAPs’ temporal characteristics reflect stress dimension of CPP patients

**Figure 4:**
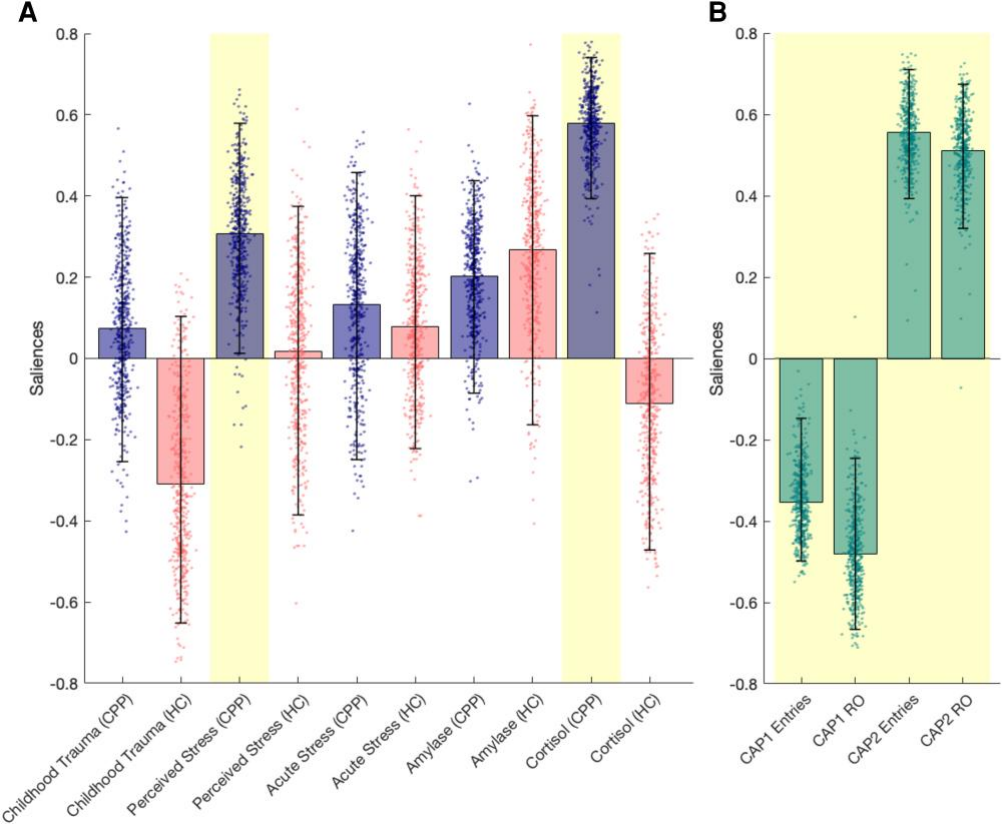
**PLSC analysis on stress-related measures (A) and temporal characteristics (B) in CPP patients (*N* = 27) and HCs (*N* = 30).** Salience weights of significant PLSC component (*P* = 0.002) are presented for stress measures and temporal characteristics. The height of each bar represents the magnitude of the variable's contribution to the multivariate correlation pattern (salience weight). The error bars reflect the 5th to 95th percentile range of the bootstrap distribution. Yellow shadings indicate salience weights that were both statistically significant and robust, i.e. stable across bootstrap resamples (dots). As the data were standardized, these weights can be interpreted in a similar way to correlation coefficients: bars pointing in the same direction indicate a positive association, while those pointing in opposite directions reflect an inverse association. Childhood trauma was measured using the childhood trauma questionnaire; perceived stress with the perceived stress scale; Amylase refers to salivary alpha-amylase concentration [U/ml]; Cortisol refers to CAR AUC_I_. CAP1/2 Entries represent the relative number of entries into CAP1 or CAP2; CAP1/2 RO indicates the relative occurrence of CAP1 or CAP2. Abbreviations: CAP = co-activation pattern, CAR AUC_I_ = cortisol awakening response calculated with the area under the curve with respect to the increase, CPP = chronic primary pain, HC = healthy control, PLSC = Partial Least Squares Correlation.

To examine the relationship between the temporal characteristics of CAPs and stress-related domains in 27 CPP patients and 30 HCs, a PLSC analysis was conducted. The analysis revealed a significant multivariate association between CAP temporal dynamics and stress-related measures (*P* = 0.002; Fig. 4, Supplementary Figs 6 and 7, Supplementary Table 4). As shown in Fig. 4, higher PSS scores and greater CAR AUC_I_ in CPP patients, but not in HCs, were related to lower relative entries and occurrences of CAP1 and higher relative entries and occurrences of CAP2. In contrast, a separate PLSC analysis and univariate correlation analysis assessing the relationship between CAP temporal characteristics and pain-related domains in CPP patients did not reveal any significant associations (Supplementary Figs 8–10).

## Discussion

A multimodal framework was used to investigate dynamic brain states involved in the stress response and pain processing in CPP, focusing on the aIC. Consistent with our hypothesis, altered temporal characteristics of brain network function were identified in CPP patients compared to HCs. These alterations were particularly evident in the co-activation of the aIC with the DMN, ECN, salience/ventral attention and somatomotor networks. Notably, these altered temporal dynamics were significantly associated with stress-related, but not pain-related measures in CPP patients, whereas no such associations were observed in HCs.

### Network coordination and coupling in CPP

By capturing transient fluctuations in brain activity, CAP analysis may provide insight into how brief, localized bursts of neural activity may propagate across networks in a cascade-like manner.^53^ Disruptions in CAP dynamics may reflect impaired coordination within or between brain networks. The observed altered temporal dynamics of aIC-seeded brain states in CPP may reflect impaired network coordination, leading to altered flexibility in switching between large-scale networks. This altered flexibility could be mediated by the aIC, as a central hub for coordinating dynamic interactions among the salience network, DMN and ECN,^14,54,55^ all of which are frequently reported to be altered in chronic pain conditions.^56^

Considering the observed altered temporal dynamics of aIC-seeded CAPs in the context of network coupling mechanisms may enhance the interpretability. We observed reduced relative entries and relative occurrence in CAP1 in CPP patients (Fig. 3), indicating reduced coupling of the aIC with both the DMN and the ECN, alongside increased coupling between the aIC and the visual network and dorsal attention network in CPP patients. These findings suggest an altered aIC connectivity profile in CPP characterized by decreased integration with networks involved in internal regulation (i.e. interoception and self-referential thinking) and executive control, alongside enhanced engagement with networks supporting externally oriented attention and visual processing.^57^ This pattern may reflect disrupted salience attribution^58^ and impaired top-down modulation,^59^ suggesting that the brain of CPP patients may struggle to appropriately prioritize pain-related signals and exert cognitive control over pain perception. In CAP2, we observed higher levels of relative occurrences and relative entries in CPP (Fig. 3), indicating increased coupling of the aIC with the salience/ventral attention network and somatomotor network, and reduced coupling with the DMN. These findings indicate heightened engagement of the aIC with networks involved in detecting salient stimuli, processing somatosensory information and motor control, alongside reduced integration with the internal regulation in patients with CPP.^57^ This pattern may reflect heightened pain vigilance and reduced capacity for internally directed regulation, such as mind-wandering or cognitive reappraisal,^60-64^ all of which may be disrupted in chronic pain.^9^

Together, these findings suggest that altered aIC-centred network dynamics in CPP reflect a shift away from internally regulated, self-referential brain states toward externally oriented, pain-focused processing, potentially contributing to the persistence of chronic pain.

### CAPs in chronic pain

Direct comparisons with previous CAP studies in chronic pain are limited by methodological and sample differences.^25,26^ On the one hand, Mawla *et al*.^25^ investigated patients with fibromyalgia, a condition classified under CPP, and showed that lower occurrence of CAPs involving salience/dorsal attention networks during sustained pain conditions was associated with higher clinical pain interference (i.e. broader impact on functioning) in patients with fibromyalgia. Additionally, the greater occurrence of CAPs involving sensorimotor network co-activation during sustained pain conditions was associated with higher clinical pain interference and greater pressure pain sensitivity (measured via cuff pressure) in patients with fibromyalgia. On the other hand, Liu *et al*.^26^ investigated women with primary dysmenorrhea (i.e. a form of chronic visceral pain, classified under chronic secondary pain) and identified changed temporal dynamics of CAPs (e.g. relative occurrence and duration), compared to HCs, involving regions of the sensorimotor, visual and salience networks. These identified alterations were associated with pain catastrophizing and menstrual pain intensity of the tested women.^26^ In contrast to these two studies, we did not observe any significant associations between CAP temporal characteristics and pain-related measures in patients with CPP.

We hypothesize that the differences in patient population may partly explain the absence of associations between brain states involving salience, frontoparietal and somatomotor networks and pain intensity in our CPP study. Additionally, Mawla *et al*. examined CAP occurrence in fibromyalgia during an induced pain condition relative to the rest condition, capturing momentary pain responses in fibromyalgia rather than pure resting-state dynamics under baseline conditions. This methodological distinction may further contribute to discrepancies in brain–pain coupling across studies. Therefore, in CPP, perceived pain may no longer be directly linked to ongoing nociceptive input but may instead be increasingly influenced by stress-related mechanisms, potentially leading to a decoupling between subjective pain reports and transient brain state dynamics. Interestingly, despite this difference in brain-pain coupling, both CPP and chronic secondary pain appear to share alterations in the temporal dynamics of certain brain states, particularly involving salience and somatomotor networks. This suggests that certain CAP features may reflect general characteristics of the chronic pain state, regardless of the underlying pain type, while their clinical expression (e.g. associations with pain intensity) may diverge depending on the mechanisms driving the pain.

Taken together, our and others’ findings suggest that while altered transient brain state dynamics are a consistent feature of patients with chronic pain, the presence or absence of associations with pain-related measures may depend on differences in patient populations, type and timing of pain assessment, or methodological aspects of CAP analysis (seed-based versus whole brain).

### Pain and stress regulation in chronic pain

In previous research on chronic pain, an increased connectivity between the DMN, the salience network and the sensorimotor network has frequently been observed and has often been associated with increased pain sensitivity.^9,65^ However, in our study, the CPP-specific connectivity patterns were not associated with pain-related measures but instead showed significant correlations with stress-related variables, including both the CAR and self-reported perceived stress (Fig. 4). In contrast, acute stress reactivity, measured as salivary alpha-amylase and self-reported acute stress load, and self-reported exposure to childhood trauma, which have previously been linked to chronic pain,^66-68^ was not associated with CPP-specific CAP dynamics in our study. Group differences (unadjusted for covariates) were observed in CAR, whereas alpha-amylase levels remained unchanged, consistent with our finding that CPP-specific CAP dynamics were associated with CAR but not with alpha-amylase. This dissociation may suggest that chronic pain affects stress regulation more strongly through the HPA axis than through acute ANS reactivity. This possibly reflects a blunted autonomic reactivity due to long-term adaptation in chronic pain, or the high variability of alpha-amylase concentrations. Taken together, our results suggest that altered brain dynamics in CPP, particularly involving the aIC, may be more closely associated with chronic stress response system activity than with acute stress responses, past trauma, and clinical pain characteristics such as pain sensitivity and intensity.

Further, the observed group differences in cortisol concentration may indicate HPA axis dysregulation, which can disrupt immune signalling and lead to stress- and inflammation-mediated central nervous system alterations that sensitize pain pathways and amplify pain perception both at the central and peripheral levels.^69^

Cortisol levels related to stress physiology have been linked to alterations in brain network connectivity, suggesting that stress response system activity may shape functional interactions within pain-related brain regions. For example, Vachon-Presseau *et al*.^70^ showed that individuals with a higher stress response, measured by salivary cortisol, exhibited reduced functional connectivity between pain-related activity in the anterior midcingulate cortex and brainstem regions, specifically the midbrain and the rostral ventromedial medulla. This effect was observed in both chronic pain patients and HCs, indicating a general modulatory effect of stress on pain-related circuits rather than a chronic pain-specific phenomenon. In contrast, our results show that associations between the temporal dynamics of CAPs and stress-related variables were only found in patients with CPP. This suggests that the observed alterations are not simply a consequence of elevated stress response levels in CPP but reflect CPP-specific pain mechanisms that interact with stress response system activity and potentially adaptive functional neuroplasticity in patients with CPP.

Taking a wider perspective, these findings align with the concept of allostatic load, which refers to the cumulative burden of chronic stress on the body’s regulatory system.^71^ Rather than maintaining a fixed set point, the brain dynamically adjusts internal states (psychological and physiological) through allostasis, a predictive process aimed at maintaining stability in response to changing internal and external demands.^72^ In chronic pain, the persistent activation of stress and pain-related systems may disrupt the regulatory balance between adaptation and recovery, potentially leading to allostatic overload, a state in which the brain’s predictive regulation becomes less efficient at adapting to chronic stress.^73^ Within these frameworks, our findings suggest that altered regulation of the CAR and elevated perceived stress load are associated with changes in aIC-seeded brain network dynamics specific to CPP. This supports the idea that adaptive stress-system activity may contribute to disrupted temporal brain patterns, reflecting a potential neurophysiological correlate of allostatic dysregulation in CPP.

Overall, our findings suggest that interactions between chronic stress physiology and aIC-centred brain dynamics may represent a key mechanism linking stress-system dysregulation to altered network function in CPP.

### Limitations

This study has several limitations that should be considered when interpreting the findings. The sample size was relatively small and included a heterogeneous group of patients with CPP in terms of pain duration and the number of pain sites. In addition, some participants were taking psychotropic medication and/or presented with psychiatric comorbidities. Although we accounted for both medication use and psychiatric comorbidities in the analyses, residual effects on brain function cannot be completely excluded. Moreover, affective symptoms and intake of psychotropic medication may partly reflect downstream consequences of CPP and could therefore be part of a causal pathway between CPP and brain dynamics. Accordingly, covariate adjustment clarifies group differences beyond these factors but may yield conservative estimates of the total condition-related differences if part of the effect operates through them. Furthermore, as this is a cross-sectional study, no causal conclusions can be drawn. Finally, the comparison group is, as in prior studies, HC, hence not providing information about CPP compared to chronic secondary pain.

Methodologically, seed-based CAP analysis comes with certain challenges. Co-activations with the seed region may occur without direct functional relevance and can be sensitive to noise, and their relationship to clinical characteristics would benefit from further experimental testing. We cannot exclude that observed CAP dynamic group differences reflect broader network-level changes rather than aIC-specific coupling changes. Seed-based CAP analysis also omits ∼70–80% of frames, which is typical for this approach,^21^ but should be considered when interpreting the results. The number of CAP clusters was selected based on consensus clustering, a common approach in the field. Nevertheless, CAP stability would likely improve with a larger sample size. Additionally, the application of PCA as a dimensionality reduction step may have reduced the visibility of weaker co-activation networks while emphasizing more dominant ones. However, previous work has shown that the PCA step offers a useful trade-off between reducing computational demands and the potential loss of weaker networks.^24^ Furthermore, our CAP results may depend on specific choices regarding preprocessing and analysis, including the use of global signal regression (GSR) and selected CAP settings (e.g. thresholding, clustering settings, CAP assignment criteria). As these choices can influence CAP spatial and temporal properties, future work should assess reproducibility across alternative preprocessing pipelines (including analyses with and without GSR) and CAP settings. Finally, translating resting-state CAP results into specific brain functions or behavioural processes remains a challenge, since it is not a direct measure of brain function.^22^

Conceptually, although the aIC is a key region in chronic pain, it represents only one node in a broader network. Our focus on the aIC was guided by its role in salience detection, and integration of sensory, emotional and cognitive aspects of pain, yet future work should also consider posterior insula and other relevant regions. Additionally, given the small sample size, the non-significant brain–pain associations in the PLSC analysis should be interpreted cautiously and may reflect limited power to detect small-to-moderate effects, rather than evidence for the absence of a brain–pain relationship. Finally, the body mass index was not collected and/or included as a covariate and should be assessed in future studies, as it may be associated with individual cortisol levels. Moreover, salivary cortisol, while widely used, reflects HPA-axis activity only indirectly and remains debated in its interpretability.^74^

## Conclusion

By integrating CAP analysis with clinical and endocrine markers, we identified a potential neural signature of altered stress processing in patients with CPP. Patients with CPP exhibited altered temporal dynamics of large-scale brain networks compared to sex- and age-matched HCs, with significant associations emerging with stress-related measures. This suggests that anterior insula–seeded brain dynamics—within a region implicated in salience detection, and emotional regulation—may be more closely linked to the stress-response system activity than to pain-related measures in CPP.

Together, these findings highlight the relevance of considering stress-related processes when interpreting neurobiological alterations in CPP. However, longitudinal and interventional studies will be essential to determine the directionality of these associations and to explore whether targeted modulation of the stress response system—at the neuronal, endocrinological and psychophysiological levels—could provide novel treatment avenues for individuals with CPP.

## Supplementary Material

fcag121_Supplementary_Data

## Data Availability

The data are not publicly available but can be shared upon request. The TbCAPs toolbox is publicly available at https://github.com/MIPLabCH/TbCAPs, and the PLS Toolbox can be accessed at https://github.com/FND-ResearchGroup/myPLS_SL. Additional codes used for data analysis are available at https://github.com/FND-ResearchGroup/CAP_in_CPP.

## References

[1] World Health Organization . ICD-11 for Mortality and Morbidity Statistics. February 2022. Accessed November 17, 2022. https://icd.who.int/browse11/l-m/en#/http%3a%2f%2fid.who.int%2ficd%2fentity%2f1581976053

[2] Castejón J, Chen F, Yasoda-Mohan A, Ó Sé C, Vanneste S. Chronic pain—A maladaptive compensation to unbalanced hierarchical predictive processing. NeuroImage. 2024;297:120711. 38942099 10.1016/j.neuroimage.2024.120711

[3] Gatchel RJ, Peng YB, Peters ML, Fuchs PN, Turk DC. The biopsychosocial approach to chronic pain: Scientific advances and future directions. Psychol Bull. 2007;133:581–624. 17592957 10.1037/0033-2909.133.4.581

[4] Treede RD, Rief W, Barke A, et al Chronic pain as a symptom or a disease: The IASP Classification of Chronic Pain for the International Classification of Diseases (ICD-11). PAIN. 2019;160(1):19. 30586067 10.1097/j.pain.0000000000001384

[5] Fitzcharles MA, Cohen SP, Clauw DJ, Littlejohn G, Usui C, Häuser W. Nociplastic pain: Towards an understanding of prevalent pain conditions. Lancet. 2021;397(10289):2098–2110. 34062144 10.1016/S0140-6736(21)00392-5

[6] Borsook D, Youssef AM, Simons L, Elman I, Eccleston C. When pain gets stuck: The evolution of pain chronification and treatment resistance. PAIN. 2018;159(12):2421–2436. 30234696 10.1097/j.pain.0000000000001401 PMC6240430

[7] Cohen SP, Vase L, Hooten WM. Chronic pain: An update on burden, best practices, and new advances. Lancet. 2021;397(10289):2082–2097. 34062143 10.1016/S0140-6736(21)00393-7

[8] Bevers K, Watts L, Kishino ND, Gatchel RJ. The biopsychosocial model of the assessment, prevention, and treatment of chronic pain. US Neurol. 2016;12(2):98–104.

[9] Kaplan CM, Kelleher E, Irani A, Schrepf A, Clauw DJ, Harte SE. Deciphering nociplastic pain: Clinical features, risk factors and potential mechanisms. Nat Rev Neurol. 2024;20(6):347–363. 38755449 10.1038/s41582-024-00966-8

[10] Lupien SJ, Juster RP, Raymond C, Marin MF. The effects of chronic stress on the human brain: From neurotoxicity, to vulnerability, to opportunity. Front Neuroendocrinol. 2018;49:91–105. 29421159 10.1016/j.yfrne.2018.02.001

[11] Li X, Hu L. The role of stress regulation on neural plasticity in pain chronification. Neural Plast. 2016;2016:6402942. 28053788 10.1155/2016/6402942 PMC5178373

[12] Abdallah CG, Geha P. Chronic pain and chronic stress: Two sides of the same coin? Chronic Stress. 2017;1:2470547017704763. 10.1177/2470547017704763 PMC5546756 28795169

[13] Labrakakis C . The role of the insular cortex in pain. Int J Mol Sci. 2023;24(6):5736. 36982807 10.3390/ijms24065736 PMC10056254

[14] Menon V, Uddin LQ. Saliency, switching, attention and control: A network model of insula function. Brain Struct Funct. 2010;214(5):655–667. 20512370 10.1007/s00429-010-0262-0 PMC2899886

[15] Wiech K, Lin CS, Brodersen KH, Bingel U, Ploner M, Tracey I. Anterior insula integrates information about salience into perceptual decisions about pain. J Neurosci. 2010;30(48):16324–16331. 21123578 10.1523/JNEUROSCI.2087-10.2010 PMC6634837

[16] Ichesco E, Schmidt-Wilcke T, Bhavsar R, et al Altered resting state connectivity of the insular cortex in individuals with fibromyalgia. J Pain. 2014;15(8):815–826.e1. 24815079 10.1016/j.jpain.2014.04.007 PMC4127388

[17] Kim J, Namgung E, Lee S, et al Disturbed insular functional connectivity and its clinical implication in patients with complex regional pain syndrome. NeuroImage Clin. 2023;38:103440. 37224606 10.1016/j.nicl.2023.103440 PMC10220260

[18] Kim JH, Choi SH, Jang JH, et al Impaired insula functional connectivity associated with persistent pain perception in patients with complex regional pain syndrome. PLoS One. 2017;12(7):e0180479. 28692702 10.1371/journal.pone.0180479 PMC5503260

[19] Lu C, Yang T, Zhao H, et al Insular cortex is critical for the perception, modulation, and chronification of pain. Neurosci Bull. 2016;32(2):191–201. 26898298 10.1007/s12264-016-0016-y PMC5563738

[20] Wiech K, Jbabdi S, Lin CS, Andersson J, Tracey I. Differential structural and resting state connectivity between insular subdivisions and other pain-related brain regions. PAIN. 2014;155(10):2047–2055. 25047781 10.1016/j.pain.2014.07.009 PMC4220010

[21] Bolton TAW, Tuleasca C, Wotruba D, et al TbCAPs: A toolbox for co-activation pattern analysis. NeuroImage. 2020;211:116621. 32058000 10.1016/j.neuroimage.2020.116621

[22] Liu X, Zhang N, Chang C, Duyn JH. Co-activation patterns in resting-state fMRI signals. NeuroImage. 2018;180:485–494. 29355767 10.1016/j.neuroimage.2018.01.041 PMC6082734

[23] Michel CM, Koenig T. EEG microstates as a tool for studying the temporal dynamics of whole-brain neuronal networks: A review. NeuroImage. 2018;180:577–593. 29196270 10.1016/j.neuroimage.2017.11.062

[24] Weber S, Bühler J, Loukas S, et al Transient resting-state salience-limbic co-activation patterns in functional neurological disorders. NeuroImage Clin. 2024;41:103583. 38422831 10.1016/j.nicl.2024.103583 PMC10944183

[25] Mawla I, Huang Z, Kaplan CM, et al Large-scale momentary brain co-activation patterns are associated with hyperalgesia and mediate focal neurochemistry and cross-network functional connectivity in fibromyalgia. PAIN. 2023;164(12):2737–2748. 37751539 10.1097/j.pain.0000000000002973 PMC10652715

[26] Liu H, Su X, Shang M, et al Abnormal dynamic functional networks during pain-free periods: Resting-state co-activation pattern analysis in primary dysmenorrhea: Abnormal dynamic functional networks in primary dysmenorrhea. NeuroImage. 2025;306:121009. 39793639 10.1016/j.neuroimage.2025.121009

[27] Zhu Y, Wang Y, Yang Z, Wang L, Hu X. Endogenous cortisol-related alterations of right anterior insula functional connectivity under acute stress. J Affect Disord. 2020;274:231–238. 32469811 10.1016/j.jad.2020.05.123

[28] Spielberger C, Gorsuch R, Lushene R, Vagg P, Jacobs G. Manual for the state-trait anxiety inventory. IV. CA: Consulting Psychologists Press; 1983.

[29] Beck AT, Ward CH, Mendelson M, Mock J, Erbaugh J. An inventory for measuring depression. Arch Gen Psychiatry. 1961;4(6):561–571. 13688369 10.1001/archpsyc.1961.01710120031004

[30] Shahid A, Wilkinson K, Marcu S, Shapiro CM, eds. Leeds Sleep Evaluation Questionnaire (LSEQ). In: STOP, THAT and one hundred other sleep scales. Springer; 2012:211–213.

[31] Klinitzke G, Romppel M, Häuser W, Brähler E, Glaesmer H. Die deutsche Version des Childhood Trauma Questionnaire (CTQ)—Psychometrische Eigenschaften in einer bevölkerungsrepräsentativen Stichprobe. PPmP—Psychother · Psychosom · Med Psychol. 2012;62:47–51. 10.1055/s-0031-1295495 22203470

[32] Cohen S, Kamarck T, Mermelstein R. A global measure of perceived stress. J Health Soc Behav. 1983;24(4):385–396. 6668417

[33] Wolfe F, Clauw DJ, Fitzcharles MA, et al The American College of Rheumatology Preliminary diagnostic criteria for fibromyalgia and measurement of symptom severity. Arthritis Care Res. 2010;62(5):600–610. 10.1002/acr.20140 20461783

[34] Radbruch L, Loick G, Kiencke P, et al Validation of the German version of the brief pain inventory. J Pain Symptom Manage. 1999;18(3):180–187. 10517039 10.1016/s0885-3924(99)00064-0

[35] Egloff N, Klingler N, von Känel R, et al Algometry with a clothes peg compared to an electronic pressure algometer: A randomized cross-sectional study in pain patients. BMC Musculoskelet Disord. 2011;12:174. 21787399 10.1186/1471-2474-12-174 PMC3158560

[36] Cámara RJA, Merz C, Wegmann B, Stauber S, von Känel R, Egloff N. Cost-saving early diagnosis of functional pain in nonmalignant pain: A noninferiority study of diagnostic accuracy. Pain Res Treat. 2016;2016:5964250. 27088013 10.1155/2016/5964250 PMC4819101

[37] Golden SH, Wand GS, Malhotra S, Kamel I, Horton K. Reliability of hypothalamic–pituitary–adrenal axis assessment methods for use in population-based studies. Eur J Epidemiol. 2011;26(7):511–525. 21533585 10.1007/s10654-011-9585-2 PMC3697932

[38] Wust S, Wolf J, Hellhammer DH, Federenko I, Schommer N, Kirschbaum C. The cortisol awakening response—Normal values and confounds. Noise Health. 2000;2(7):79–88. 12689474

[39] Stalder T, Kirschbaum C, Kudielka BM, et al Assessment of the cortisol awakening response: Expert consensus guidelines. Psychoneuroendocrinology. 2016;63:414–432. 26563991 10.1016/j.psyneuen.2015.10.010

[40] Ali N, Nater UM. Salivary alpha-amylase as a biomarker of stress in behavioral medicine. Int J Behav Med. 2020;27(3):337–342. 31900867 10.1007/s12529-019-09843-x PMC7250801

[41] Pruessner JC, Kirschbaum C, Meinlschmid G, Hellhammer DH. Two formulas for computation of the area under the curve represent measures of total hormone concentration versus time-dependent change. Psychoneuroendocrinology. 2003;28(7):916–931. 12892658 10.1016/s0306-4530(02)00108-7

[42] Power JD, Barnes KA, Snyder AZ, Schlaggar BL, Petersen SE. Spurious but systematic correlations in functional connectivity MRI networks arise from subject motion. NeuroImage. 2012;59(3):2142–2154. 22019881 10.1016/j.neuroimage.2011.10.018 PMC3254728

[43] Liu X, Duyn JH. Time-varying functional network information extracted from brief instances of spontaneous brain activity. Proc Natl Acad Sci U S A. 2013;110(11):4392–4397. 23440216 10.1073/pnas.1216856110 PMC3600481

[44] Zhang L, Luo L, Zhou Z, et al Functional connectivity of anterior Insula predicts recovery of patients with disorders of consciousness. Front Neurol. 2018;9:1024. 30555407 10.3389/fneur.2018.01024 PMC6283978

[45] Monti S, Tamayo P, Mesirov J, Golub T. Consensus clustering: A resampling-based method for class discovery and visualization of gene expression microarray data. Mach Learn. 2003;52(1):91–118.

[46] Șenbabaoğlu Y, Michailidis G, Li JZ. Critical limitations of consensus clustering in class discovery. Sci Rep. 2014;4(1):6207. 25158761 10.1038/srep06207 PMC4145288

[47] Thomas Yeo BT, Krienen FM, Sepulcre J, et al The organization of the human cerebral cortex estimated by intrinsic functional connectivity. J Neurophysiol. 2011;106(3):1125–1165. 21653723 10.1152/jn.00338.2011 PMC3174820

[48] Weber S, Bühler J, Bolton TAW, Aybek S. Altered brain network dynamics in motor functional neurological disorders: The role of the right temporo-parietal junction. Transl Psychiatry. 2025;15(1):167. 40374624 10.1038/s41398-025-03385-5 PMC12081630

[49] Griffa A, Bommarito G, Assal F, Herrmann FR, Van De Ville D, Allali G. Dynamic functional networks in idiopathic normal pressure hydrocephalus: Alterations and reversibility by CSF tap test. Hum Brain Mapp. 2021;42(5):1485–1502. 33296129 10.1002/hbm.25308 PMC7927299

[50] Zöller D, Sandini C, Karahanoğlu FI, et al Large-scale brain network dynamics provide a measure of psychosis and anxiety in 22q11.2 deletion syndrome. Biol Psychiatry Cogn Neurosci Neuroimaging. 2019;4(10):881–892. 31171499 10.1016/j.bpsc.2019.04.004

[51] Krishnan A, Williams LJ, McIntosh AR, Abdi H. Partial least squares (PLS) methods for neuroimaging: A tutorial and review. NeuroImage. 2011;56(2):455–475. 20656037 10.1016/j.neuroimage.2010.07.034

[52] McIntosh AR, Lobaugh NJ. Partial least squares analysis of neuroimaging data: Applications and advances. NeuroImage. 2004;23:S250–S263. 15501095 10.1016/j.neuroimage.2004.07.020

[53] Tagliazucchi E, Balenzuela P, Fraiman D, Chialvo DR. Criticality in large-scale brain fMRI dynamics unveiled by a novel point process analysis. Front Physiol. 2012;3:15. 22347863 10.3389/fphys.2012.00015 PMC3274757

[54] Molnar-Szakacs I, Uddin LQ. Anterior insula as a gatekeeper of executive control. Neurosci Biobehav Rev. 2022;139:104736. 35700753 10.1016/j.neubiorev.2022.104736

[55] Ferraro S, Klugah-Brown B, Tench CR, et al Dysregulated anterior insula reactivity as robust functional biomarker for chronic pain—Meta-analytic evidence from neuroimaging studies. Hum Brain Mapp. 2022;43(3):998–1010. 34734458 10.1002/hbm.25702 PMC8764475

[56] Dhanaraj V, Rolfe NW, Dadario NB, et al Multi-network dynamical structure of the human brain in the setting of chronic pain: A coordinate-based meta-analysis. Brain Commun. 2025;7(5):fcaf343. 41169268 10.1093/braincomms/fcaf343 PMC12569763

[57] Fernandez Z, Scheel N, Baker JH, Zhu DC. Functional connectivity of cortical resting-state networks is differentially affected by rest conditions. Brain Res. 2022;1796:148081. 36100086 10.1016/j.brainres.2022.148081

[58] Rehm S, Sachau J, Hellriegel J, et al Pain matters for central sensitization: Sensory and psychological parameters in patients with fibromyalgia syndrome. Pain Rep. 2021;6(1):e901. 33718743 10.1097/PR9.0000000000000901 PMC7952123

[59] Schrepf A, Moser S, Harte SE, et al Top down or bottom up? An observational investigation of improvement in fibromyalgia symptoms following hip and knee replacement. Rheumatology. 2020;59(3):594–602. 31411333 10.1093/rheumatology/kez303 PMC7998337

[60] Zhang J, Wang H, Guo L. Investigating the brain functional abnormalities underlying pain hypervigilance in chronic neck and shoulder pain: A resting-state fMRI study. Neuroradiology. 2024;66(8):1353–1361. 38296904 10.1007/s00234-024-03286-2

[61] Kucyi A, Salomons TV, Davis KD. Mind wandering away from pain dynamically engages antinociceptive and default mode brain networks. Proc Natl Acad Sci U S A. 2013;110(46):18692–18697. 24167282 10.1073/pnas.1312902110 PMC3832014

[62] Vachon-Presseau E, Centeno MV, Ren W, et al The emotional brain as a predictor and amplifier of chronic pain. J Dent Res. 2016;95(6):605–612. 26965423 10.1177/0022034516638027 PMC4924545

[63] Yu L, Norton S, McCracken LM. Change in ‘self-as-context’ (‘perspective-taking’) occurs in acceptance and commitment therapy for people with chronic pain and is associated with improved functioning. J Pain. 2017;18(6):664–672. 28131700 10.1016/j.jpain.2017.01.005

[64] Koechlin H, Coakley R, Schechter N, Werner C, Kossowsky J. The role of emotion regulation in chronic pain: A systematic literature review. J Psychosom Res. 2018;107:38–45. 29502762 10.1016/j.jpsychores.2018.02.002

[65] Cavicchioli M, Scalabrini A, Nimbi F, et al Fibromyalgia and the painful self: A meta-analysis of resting-state fMRI data. J Psychiatr Res. 2025;183:61–71. 39938202 10.1016/j.jpsychires.2025.01.048

[66] Coppens E, Kempke S, Van Wambeke P, et al Cortisol and subjective stress responses to acute psychosocial stress in fibromyalgia patients and control participants. Psychosom Med. 2018;80(3):317. 29232329 10.1097/PSY.0000000000000551

[67] Shirasaki S, Fujii H, Takahashi M, et al Correlation between salivary α-amylase activity and pain scale in patients with chronic pain. Reg Anesth Pain Med. 2007;32(2):120–123. 17350522 10.1016/j.rapm.2006.11.008

[68] Nicolson KP, Mills SEE, Senaratne DNS, Colvin LA, Smith BH. What is the association between childhood adversity and subsequent chronic pain in adulthood? A systematic review. BJA Open. 2023;6:100139. 37588177 10.1016/j.bjao.2023.100139 PMC10430872

[69] Hannibal KE, Bishop MD. Chronic stress, cortisol dysfunction, and pain: A psychoneuroendocrine rationale for stress management in pain rehabilitation. Phys Ther. 2014;94(12):1816–1825. 25035267 10.2522/ptj.20130597 PMC4263906

[70] Vachon-Presseau E, Martel MO, Roy M, et al Acute stress contributes to individual differences in pain and pain-related brain activity in healthy and chronic pain patients. J Neurosci. 2013;33(16):6826–6833. 23595741 10.1523/JNEUROSCI.4584-12.2013 PMC6618876

[71] McEwen BS, Karatsoreos IN. Sleep deprivation and circadian disruption: Stress, allostasis, and allostatic load. Sleep Med Clin. 2015;10(1):1–10. 26055668 10.1016/j.jsmc.2014.11.007 PMC8935364

[72] Sterling P . Allostasis: A model of predictive regulation. Physiol Behav. 2012;106(1):5–15. 21684297 10.1016/j.physbeh.2011.06.004

[73] Bonanno M, Papa D, Cerasa A, Maggio MG, Calabrò RS. Psycho-neuroendocrinology in the rehabilitation field: Focus on the complex interplay between stress and pain. Medicina (Mex). 2024;60(2):285. 10.3390/medicina60020285 PMC10889914 38399572

[74] Hellhammer DH, Wüst S, Kudielka BM. Salivary cortisol as a biomarker in stress research. Psychoneuroendocrinology. 2009;34(2):163–171. 19095358 10.1016/j.psyneuen.2008.10.026

