# Supplementary material for: Anterior insular co-activation patterns associated with stress markers in chronic primary pain
Source: Brain Commun. 2026 Apr 3;8(2):fcag121. doi: 10.1093/braincomms/fcag121 (PMC13070617; doi:10.1093/braincomms/fcag121)
Supplement: fcag121_Supplementary_Data [file fcag121_supplementary_data.pdf]

SUPPLEMENTARY MATERIAL:
Anterior insular co-activation patterns associated with stress markers in chronic primary pain

Salome Häuselmann[1,2,3], Anna Wyss[1,3], Samantha Weber[1,4,5], Nicolas Gninenko[1,6], Cristina Concetti[6], Eliane Müller[1,2,6], Rupert Bruckmaier[7], Josef Gross[7], Nina Bischoff[1], Chantal Berna[8], Martin grosse Holtforth[1,9*] & Selma Aybek[6*]

Author affiliations:
1 Psychosomatic Medicine, Department of Neurology, Inselspital, Bern University Hospital, University of Bern, Switzerland
2 Graduate School of Cellular and Biomedical Sciences (GCB), University of Bern, Switzerland
3 Translational Imaging Center (TIC), Swiss Institute for Translational and Entrepreneurial Medicine, Bern, Switzerland
4 Department of Adult Psychiatry and Psychotherapy, University Hospital of Psychiatry Zurich, University of Zurich, Switzerland
5 Faculty of Medicine, University of Zurich, Switzerland
6 Faculty of Science and Medicine, Department of Neurology, University of Fribourg, Switzerland
7 Veterinary Physiology, Vetsuisse Faculty, University of Bern, Switzerland
8 Center for Integrative and Complementary Medicine, Department of Anesthesiology, Lausanne University Hospital, Switzerland
9 Institute of Psychology, University of Bern, Switzerland
[*]These authors share their last authorship.

A. Anterior insular cortex (aIC) CAPs derived from healthy controls (HCs)

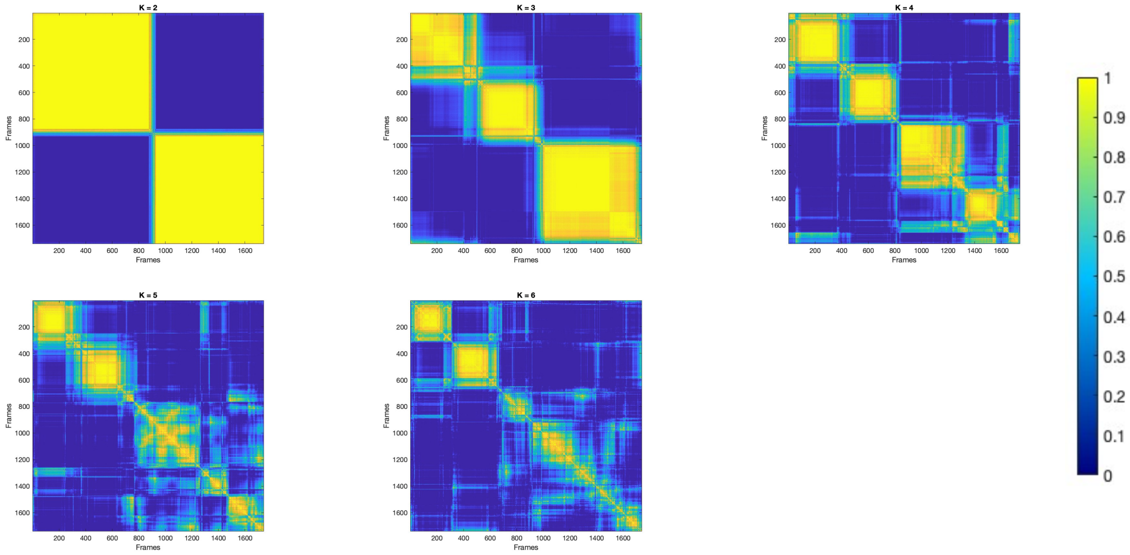

Supplementary Fig. 1. Consensus matrices (C_k) from aIC CAPs analysis. Consensus matrices were computed for each cluster number k to assess the stability of the clustering. For each k, k-means clustering was repeated over 200 folds, each time on a randomly selected subsample containing 80% of the data (without replacement). For any given pair of data points i and j (i.e., two retained fMRI volumes), the consensus value represents the proportion of folds in which both i and j were included and assigned to the same cluster. These values were averaged across the relevant folds and stored in the consensus matrix C_k. High consensus values indicate that a pair was consistently assigned to the same cluster (i.e., yellow), while low values reflect inconsistent assignments (i.e., in blue shades). Stable clustering solutions are characterized by sharp, well-defined boundaries in the consensus matrix, indicating robust groupings. In contrast, diffuse boundaries suggest less stable or ambiguous clustering.

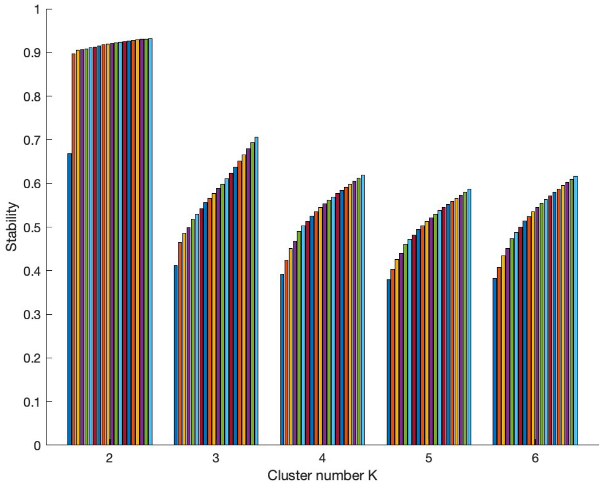

Supplementary Fig. 2. Stability measure (1 − PAC) derived from the aIC CAPs analysis. This measure is based on the cumulative distribution of consensus values across all pairs of data points (i.e., upper diagonal of the consensus matrix C_k). The distribution is denoted as $P_k(c)$, where $c \in [0, 1]$ indicates the consensus value. The proportion of ambiguously clustered pairs (PAC) is defined as the fraction of consensus values that fall within an intermediate range, bounded by a threshold c_t, and reflect insufficiently consistent assignments across cross-validation folds. Lower PAC values and correspondingly higher 1−PAC values indicate more stable and robust clustering solutions. Colored bars represent results obtained using varying c_t thresholds.

Supplementary Table 1. Imaging data of patients with CPP and HCs.

	**CPP (N = 30)**	**HC (N = 30)**	**Statistics [a]**
Retained Frames (mean, SD)	56.7 (9.35)	58.0 (5.06)	W = 477, ns
Excluded Frames [b] (mean, SD)	3.17 (6.05)	1.23 (3.39)	W = 538.5, ns

[a] Wilcoxon rank-sum test. Significance code: ***p < 0.001, **p < 0.01, *p < 0.05. SD: standard deviation, ns: not significant. [b] Frames above the threshold (0.84 SD) that were scrubbed, based on Power's framewise displacement criterion.

Supplementary Table 2. AAL3-defined regions contributing to CAPs.

	**Regions positive contribution**	Peak MNI coordinates	**Regions negative contribution**	Peak MNI coordinates
**CAP1**	Frontal_Sup_Medial_L	-2, 34, 40	Precuneus_R	8, -56, 68
	Insula_L	-34, 20, -6	Fusiform_L	-30, -76, -18
	Insula_R	38, 20, -4	Temporal_Inf_R	48, -56, -18
	Frontal_Mid_2_L	-46, 18, 46	Lingual_R	18, -72, -14
	Parietal_Inf_R	50, -54, 54		
	Temporal_Mid_L	-62, -30, -6		
	Frontal_Sup_2_R	30, 54, 14		
	Frontal_Mid_2_R	42, 22, 42		
	Caudate_L	-10, 16, 0		
	Caudate_R	12, 14, 6		
	Cingulate_Mid_L	0, -22, 42		
	Temporal_Mid_R	64, -26, -10		
	Frontal_Sup_2_R	22, 44, 36		
**CAP2**	Insula_R	34, 20, 6	Angular_L	-42, -70, 44
	Insula_L	-32, 16, 8	Cingulate_Post_L	0, 54, 24
	Supp_Motor_Area_L	-2, 6, 48	Cerebelum_Crus1_R	44, -72, -38
	Frontal_Mid_2_R	44, 40, 6	Angular_R	44, -66, 46
	Postcentral_R	26, -40, 72	Temporal_Mid_L	-66, -36, 8
	Postcentral_L	-26, -40, 68	Cerebelum_Crus2_L	-20, -90, -30
	Cingulate_Mid_L	-14, -30, 42	Frontal_Mid_2_L	-40, 18, 54
	Precuneus_L	-8, -46, 64	Temporal_Mid_R	64, -6, -20
			Rectus_R	2, 54, -18
			Cerebelum_Crus1_L	-42, -74, -36
			Rectus_L	-2, 54, -18
			Frontal_Med_Orb_L	-4, 60, -8
**CAP3**	Occipital_Sup_R	22, -86, 32	Angular_L	-42, -72, 40
	Insula_R	40, 18, -2	Precuneus_L	2, -58, 28
	Insula_L	-38, 18, 0	Angular_R	46, -68, 44
	Supp_Motor_Area_R	4, 14, 56	Frontal_Med_Orb_L	2, 58, -10
	SupraMarginal_R	62, -38, 30	Frontal_Mid_2_L	-24, 28, 52
	Frontal_Mid_2_R	48, 4, 54	Temporal_Mid_L	-66, -16, -18
	Parietal_Inf_R	26, -56, 54	Temporal_Mid_L	-66, –36, -10
	Frontal_Mid_2_R	38, 42, 32	Frontal_Sup_2_R	26, 28, 52
	Cerebelum_8_L	-22, -68, -48		
	Supp_Motor_Area_L	-2, 16, 44		
	Parietal_Sup_L	-22, -62, 58		
	Frontal_Mid_2_R	44, 40, 8		
	Parietal_Sup_L	-26, -52, 48		

Supplementary Table 3. Sensitivity analyses of group differences in CAP temporal characteristics under alternative covariate adjustment.

	**Model covariates**	**CAP1 (HC−CPP)**[a]	**CAP2 (HC−CPP)**[a]	**CAP3 (HC−CPP)** [a]
Duration	Minimal (age, sex, selected frames)	estimate = -0.04, SE = 0.10, p_{FDR} = 0.760, Cohen's *d* = -0.10	estimate = 0.02, SE = 0.10, p_{FDR} = 0.842, Cohen's *d* = 0.05	estimate = -0.06, SE = 0.10, p_{FDR} = 0.718, Cohen's *d* = -0.15
	Primary (age, sex, sum BDI and STAI2, psychotropic medication, selected frames)	estimate = -0.10, SE = 0.12, p_{FDR} = 0.513, Cohen's *d* = -0.26	estimate = -0.04, SE = 0.12, p_{FDR} = 0.744, Cohen's *d* = -0.10	estimate = -0.12, SE = 0.12, p_{FDR} = 0.488, Cohen's *d* = -0.31
	Extended (age, sex, sum BDI and STAI2, psychotropic medication, non-op analgesics, selected frames)	estimate = -0.12, SE = 0.12, p_{FDR} = 0.407, Cohen's *d* = -0.31	estimate = -0.06, SE = 0.12, p_{FDR} = 0.625, Cohen's *d* = -0.15	estimate = -0.14, SE = 0.12, p_{FDR} = 0.382, Cohen's *d* = -0.36
Relative entries	Minimal (age, sex, selected frames)	estimate = 0.06, SE = 0.02, p_{FDR} = 0.001, Cohen's *d* = 0.94	estimate = -0.07, SE = 0.02, p_{FDR} = 0.001, Cohen's *d* = -1.00	estimate = 0.02, SE = 0.02, p_{FDR} = 0.185, Cohen's *d* = 0.34
	Primary (age, sex, sum BDI and STAI2, psychotropic medication, selected frames)	estimate = 0.07, SE = 0.02, p_{FDR} = 0.002, Cohen's *d* = 1.13	estimate = -0.05, SE = 0.02, p_{FDR} = 0.022, Cohen's *d* = -0.82	estimate = 0.03, SE = 0.02, p_{FDR} = 0.16, Cohen's *d* = 0.53
	Extended (age, sex, sum BDI and STAI2, psychotropic medication, non-op analgesics, selected frames)	estimate = 0.07, SE = 0.02, p_{FDR} = 0.002, Cohen's *d* = 1.15	estimate = -0.05, SE = 0.02, p_{FDR} = 0.026, Cohen's *d* = -0.79	estimate = 0.04, SE = 0.02, p_{FDR} = 0.140, Cohen's *d* = 0.55
Relative occurrence	Minimal (age, sex, selected frames)	estimate = 0.09, SE = 0.03, p_{FDR} = 0.004, Cohen's *d* = 0.80	estimate = -0.09, SE = 0.03, p_{FDR} = 0.004, Cohen's *d* = -0.83	estimate = 0.02, SE = 0.03, p_{FDR} = 0.650, Cohen's *d* = 0.20
	Primary (age, sex, sum BDI and STAI2, psychotropic medication, selected frames)	estimate = 0.09, SE = 0.04, p_{FDR} = 0.022, Cohen's *d* = 0.82	estimate = -0.09, SE = 0.04, p_{FDR} = 0.022, Cohen's *d* = -0.80	estimate = 0.02, SE = 0.03, p_{FDR} = 0.524, Cohen's *d* = 0.23
	Extended (age, sex, sum BDI and STAI2, psychotropic medication, non-op analgesics, selected frames)	estimate = 0.09, SE = 0.04, p_{FDR} = 0.026, Cohen's *d* = 0.82	estimate = -0.09, SE = 0.04, p_{FDR} = 0.026, Cohen's *d* = -0.81	estimate = 0.02, SE = 0.04, p_{FDR} = 0.547, Cohen's *d* = 0.22

[a] Linear mixed-effects models were fitted with a group-by-state interaction as the main fixed effect and subject ID as a random intercept. Covariates included in each model are listed in the table. Post-hoc comparisons between groups were conducted within each CAP state using linear contrasts (HC − CPP). P-values from the three linear models were adjusted for multiple comparisons across models (with the same covariates) using the false discovery rate (FDR) method. The table reports the contrast estimate, standard error (SE), FDR-adjusted p-value (p_{FDR}), and Cohen's d.

CAP1:

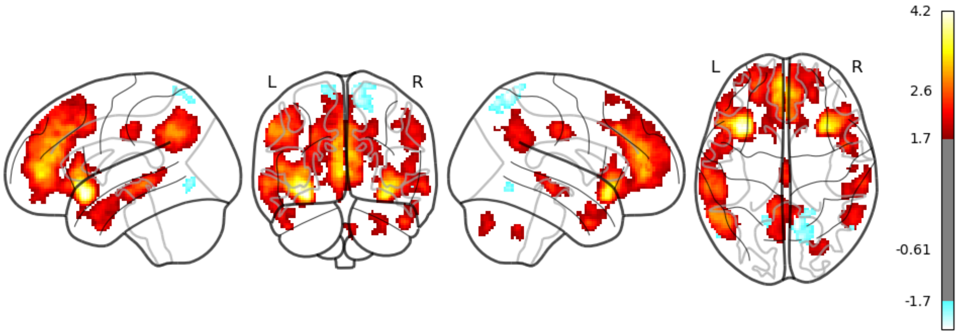

CAP 2:

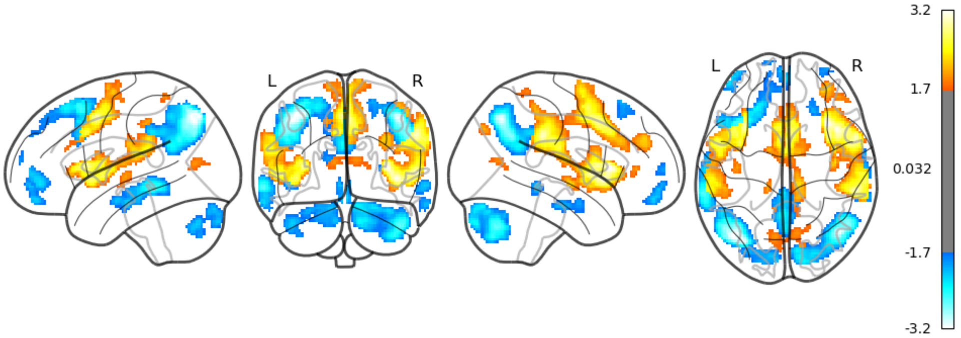

CAP3:

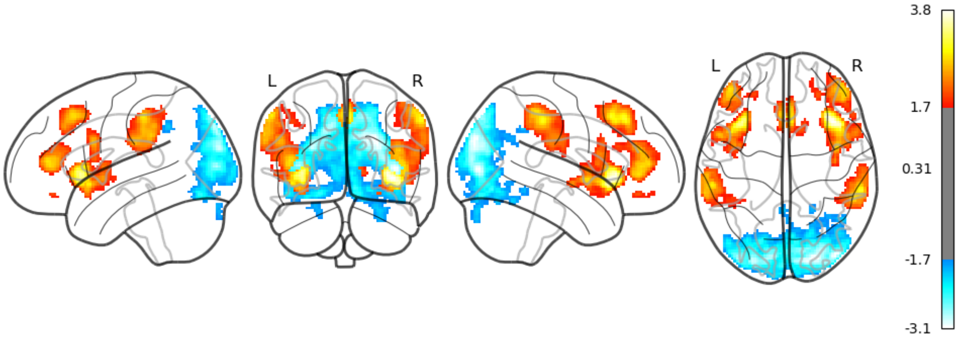

CAP4:

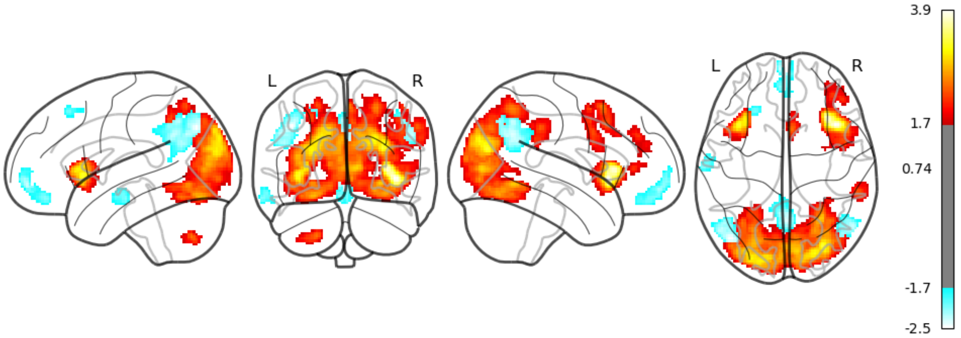

Supplementary Fig. 3. CAP maps based on aIC seed activation obtained for the second most stable cluster number (K = 4). Four CAPs were detected using HCs as the reference population (N = 30). CAPs were z-scored, and only the top 5% most positive and the top 5% most negative contributions are shown ($z > \pm 1.65$), with red indicating co-activation and blue indicating co-deactivation. The locations are shown using the standard coordinates in the Montreal Neurological Institute (MNI) space.

B. Insula CAPs derived from HCs

The insula seed was defined using the Automated Anatomical Labeling 3 (AAL3) atlas (1) to create the mask for the seed-based CAPs analysis.

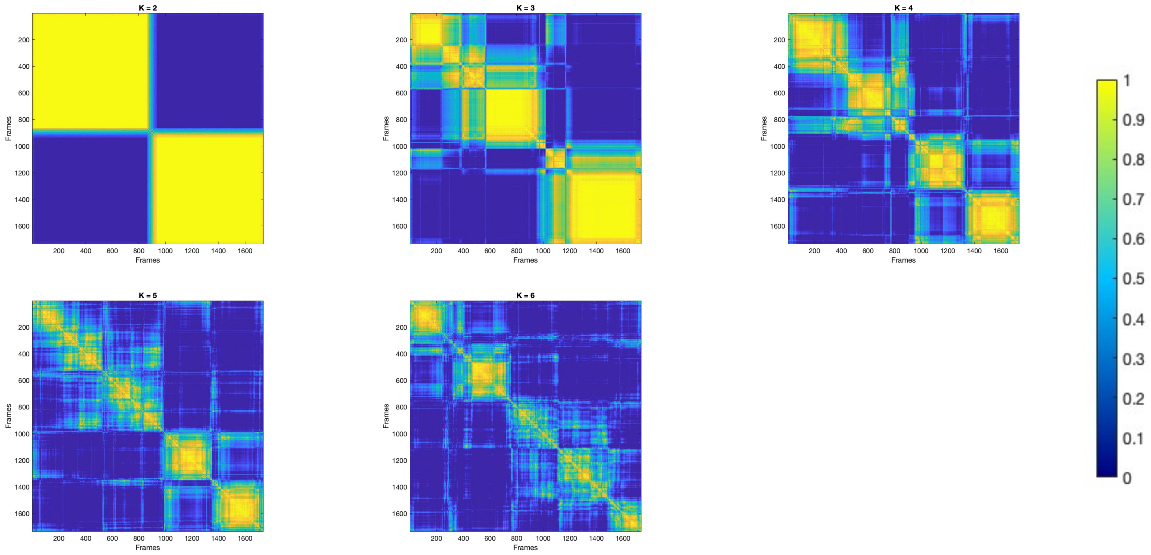

Supplementary Fig. 4. Consensus matrices (C_k) from insula CAPs analysis. Consensus matrices were computed for each cluster number k to assess the stability of the clustering. For each k, k-means clustering was repeated over 200 folds, each time on a randomly selected subsample containing 80% of the data (without replacement). For any given pair of data points i and j (i.e., two retained fMRI volumes), the consensus value represents the proportion of folds in which both i and j were included and assigned to the same cluster. These values were averaged across the relevant folds and stored in the consensus matrix C_k. High consensus values indicate that a pair was consistently assigned to the same cluster (i.e., yellow), while low values reflect inconsistent assignments (i.e., in blue shades). Stable clustering solutions are characterized by sharp, well-defined boundaries in the consensus matrix, indicating robust groupings. In contrast, diffuse boundaries suggest less stable or ambiguous clustering.

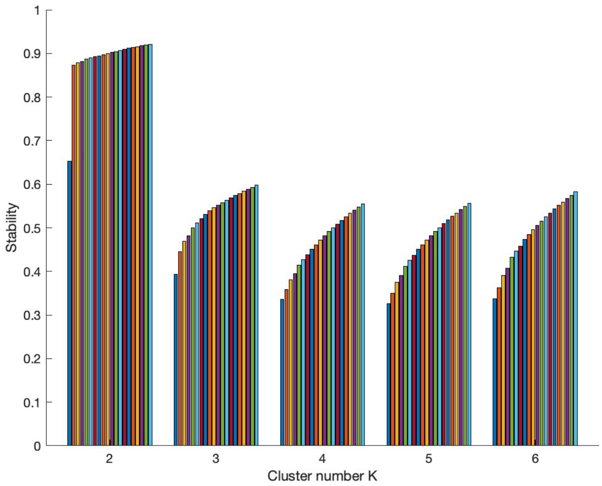

Supplementary Fig. 5. Stability measure (1 − PAC) derived from the insula CAPs analysis. This measure is based on the cumulative distribution of consensus values across all pairs of data points (i.e., upper diagonal of the consensus matrix C_k). The distribution is denoted as $P_k(c)$, where $c \in [0, 1]$ indicates the consensus value. The proportion of ambiguously clustered pairs (PAC) is defined as the fraction of consensus values that fall within an intermediate range, bounded by a threshold c_t, and reflect insufficiently consistent assignments across cross-validation folds. Lower PAC values and correspondingly higher 1−PAC values indicate more stable and robust clustering solutions. Colored bars represent results obtained using varying c_t thresholds.

C. Relationship CAPs temporal characteristics and stress-related measures in CPP and HC

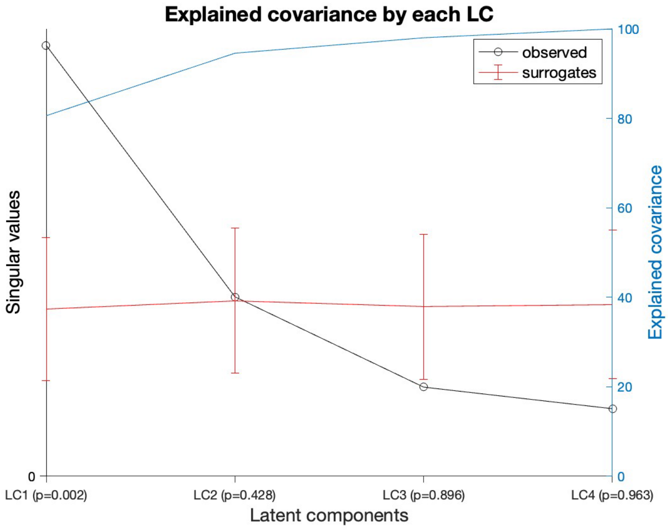

Supplementary Fig. 6. Explained covariance by each latent component (LC; i.e., optimally weighted linear combinations). Black circles represent the observed singular values for each LC, while red lines with error bars show the mean and variability of singular values obtained from permuted (surrogate) data under the null hypothesis. The blue line indicates the cumulative explained covariance across components. P-values (shown below each LC) reflect the statistical significance of each component based on permutation testing. LC1 was statistically significant (*p* = 0.002) and accounted for the largest proportion of shared variance between the datasets.

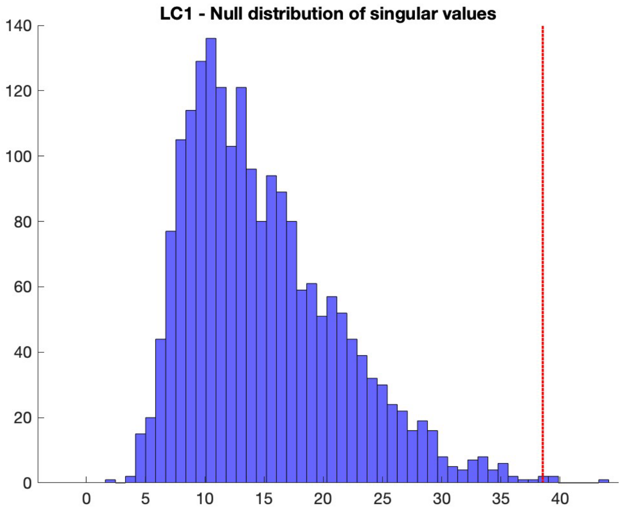

Supplementary Fig. 7. Permutation-based null distribution of singular values for the significant latent component. The histogram shows the distribution of singular values obtained from 2000 permutations under the null hypothesis of no association between the datasets. The red line indicates the observed singular value from the original (unpermuted) data. The x-axis represents singular values, and the y-axis indicates their frequency across permutations. The observed value falls outside the null distribution, indicating that the LC1 is statistically significant (*p* = 0.002).

Supplementary Table 4. Exact values of the mean bootstrap weights, along with the lower and upper bounds of the 95% confidence intervals (CIs), are reported for the statistically significant PLSC component (LC1; p = 0.002). Significant rows are shown in bold.

	Mean bootstrapped weights	Lower CI	Upper CI
Imaging Salience	**-0.392766435380866**	**0.497648362268251**	**-0.147435728688473**
	**-0.511362978338992**	**-0.666174677076771**	**-0.245648530149376**
	**0.547849267811002**	**0.392774627577017**	**0.710130301722498**
	**0.533013706560565**	**0.319965324032875**	**0.674252595328525**
Behavioral Salience	0.0807173112893264	-0.254156400948691	0.396332188626464
	**0.335042109188527**	**0.0125579075420201**	**0.579086289043699**
	0.180308282464277	-0.249184975032401	0.457748175182631
	0.273141140767941	-0.0851953400585509	0.437551252918771
	**0.684405229806786**	**0.393297797501501**	**0.740474623738921**
	-0.422636705091011	-0.651274586428179	0.103319536498072
	0.01390623637583	-0.385481364926888	0.37434137422525
	0.0961313927056307	-0.222271865317651	0.400401815432049
	0.328557888639255	-0.163168037884914	0.597313602368677
	-0.0984739756772349	-0.471872462424339	0.258393136161477

D. Relationship CAPs temporal characteristics and pain-related measures in CPP

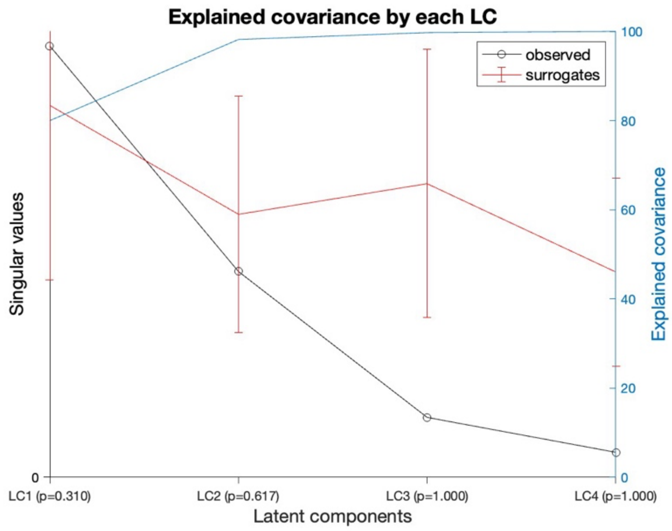

Supplementary Fig. 8. Explained covariance by each latent component (LC; i.e., optimally weighted linear combinations). Black circles represent the observed singular values for each LC, while red lines with error bars show the mean and variability of singular values obtained from permuted (surrogate) data under the null hypothesis. The blue line indicates the cumulative explained covariance across components. P-values (shown below each LC) reflect the statistical significance of each component based on permutation testing. No LC was statistically significant.

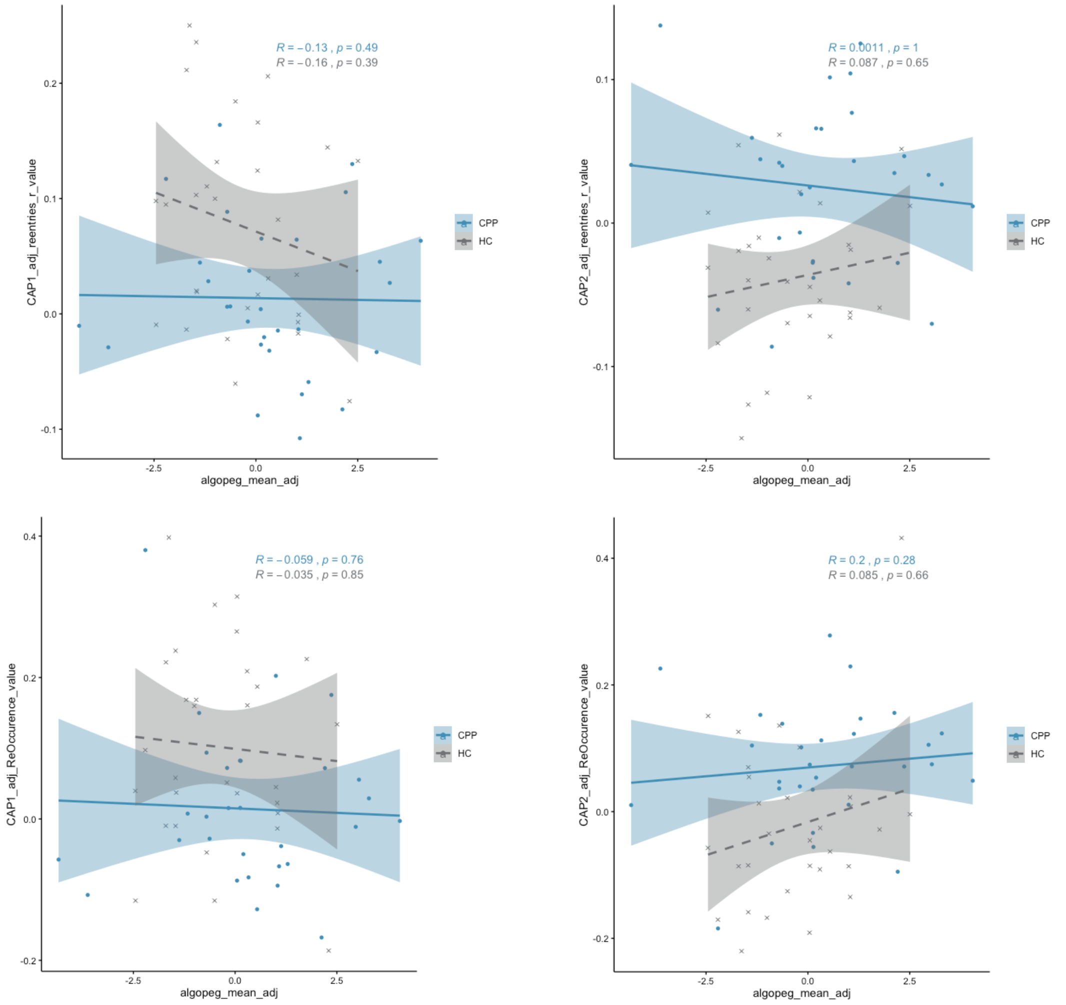

Supplementary Fig. 9. Spearman correlations between CAPs' temporal characteristics and peg algometry in CPP patients and HC (not corrected for multiple comparisons). All variables were adjusted for covariates of no interest prior to analysis. Scatterplots with fitted regression lines are shown, with R referring to Spearman's rank correlation coefficient (ρ), and p denoting the corresponding significance level. Algopeg_mean_adj = Peg algometry - mean; CAP1/2_adj_reentires_r_value = relative number of entries into CAP1 or CAP2; CAP1/2_adj_ReOccurence_value = relative occurrence of CAP1 or CAP2.

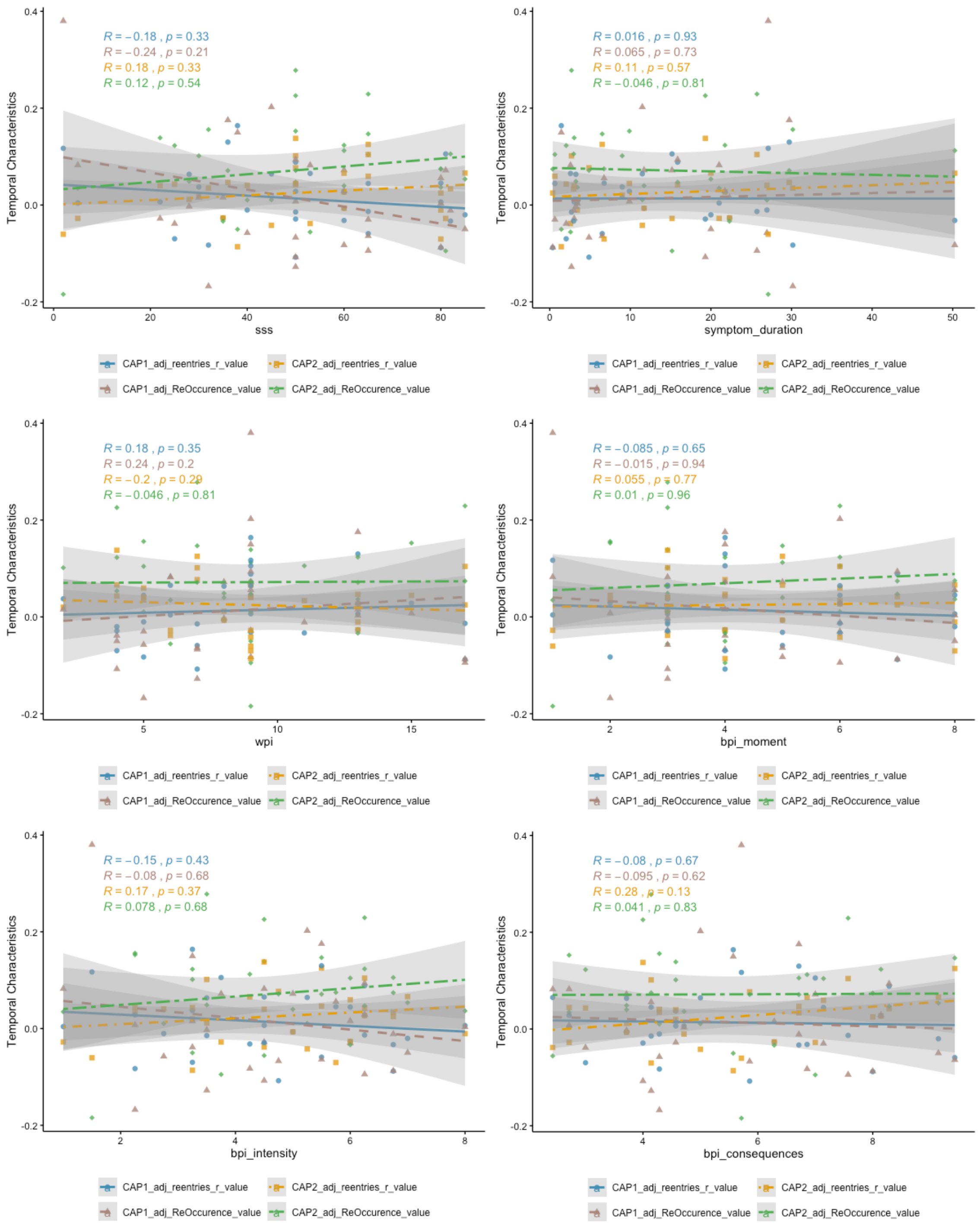

Supplementary Fig. 10. Spearman correlations between CAPs' temporal characteristics and pain-related measures in CPP patients (not corrected for multiple comparisons). Temporal characteristics were adjusted for covariates of no interest prior to analysis. Scatterplots with fitted regression lines are shown, with R referring to Spearman's rank correlation coefficient (ρ), and p denoting the corresponding significance level. sss = Subjective symptom load; sympotm_duration = symptome duration in years; wpi = widespread pain index total score; bpi_moment = pain intensity (current) score; bpi_intensity = BPI severity score; bpi_consequences = BPI interference score; CAP1/2_adj_reentires_r_value = relative number of entries into CAP1 or CAP2; CAP1/2_adj_ReOccurence_value = relative occurrence of CAP1 or CAP2.

E. Static resting-state functional connectivity of the aIC

Group differences in aIC static resting-state functional connectivity between 30 CPP patients and 30 HCs were assessed using the CONN toolbox (version 22.v2407) (2). Preprocessed data, as described in the manuscript, served as the input for this analysis.

At the first level, seed-based connectivity maps were computed to characterize the spatial pattern of functional connectivity with a predefined seed region, the aIC (mask described in the manuscript). Functional connectivity strength was quantified using Fisher-transformed bivariate correlation coefficients, derived from a weighted general linear model (weighted GLM) that estimated the association between the BOLD signal time series of the seed region and each target voxel. To account for potential transient magnetization effects at the beginning of the scanning session, individual scans were weighted using a step function convolved with the canonical hemodynamic response function (HRF) from SPM, followed by rectification.

Group-level analyses were performed using a general linear model (GLM), with separate models estimated for each voxel. First-level connectivity values served as dependent variables, and group membership as the independent variable. Age, sex, combined BDI/STAI-II sum score, and psychotropic medication status were included as covariates of no interest to control for potential confounding effects. Two group contrasts were tested to assess between-group differences in functional connectivity of 30 CPP patients and 30 HC: (1) CPP > HC, and (2) CPP < HC. Voxel-wise hypotheses were tested using multivariate parametric statistics, incorporating random effects across subjects and estimating sample covariance across multiple measurements. Statistical inference was conducted at the cluster level, based on groups of contiguous voxels. Cluster-level inference relied on Gaussian Random Field Theory. A voxel-level cluster-forming threshold of $p < 0.001$ (uncorrected) was applied, followed by a minimum cluster size threshold of 20 voxels. No correction for multiple comparisons was applied at the cluster level.

Supplementary Table 5. AAL3-defined brain regions showing significant group differences in static resting-state functional connectivity with aIC seed.

	**Regions**	Peak MNI coordinates	$\mathbf{k_E}$	Cluster-level p_{uncorr}
**CPP > HC**	Precuneus_L	0, -46, 54	38	0.015
	Frontal_Mid_2_L	-24, 32, 18	28	0.032
	OFCmed_L	18, 46, -22	22	0.053
	Frontal_Mid_2_R	34, 52, 14	20	0.064
**HC > CPP**	Thalamus_L	-22, -26, 4	46	0.008
	Cerebelum_10_R	22, 34, 42	29	0.029
	Lingual_L	-22, -64, -10	25	0.041
	Cerebelum_9_L	-2, -56, -50	22	0.997
	Cerebelum_6_L	-10, - 78, -14	20	0.064
	Temporal_Mid_R	42, -58, 0	21	0.058

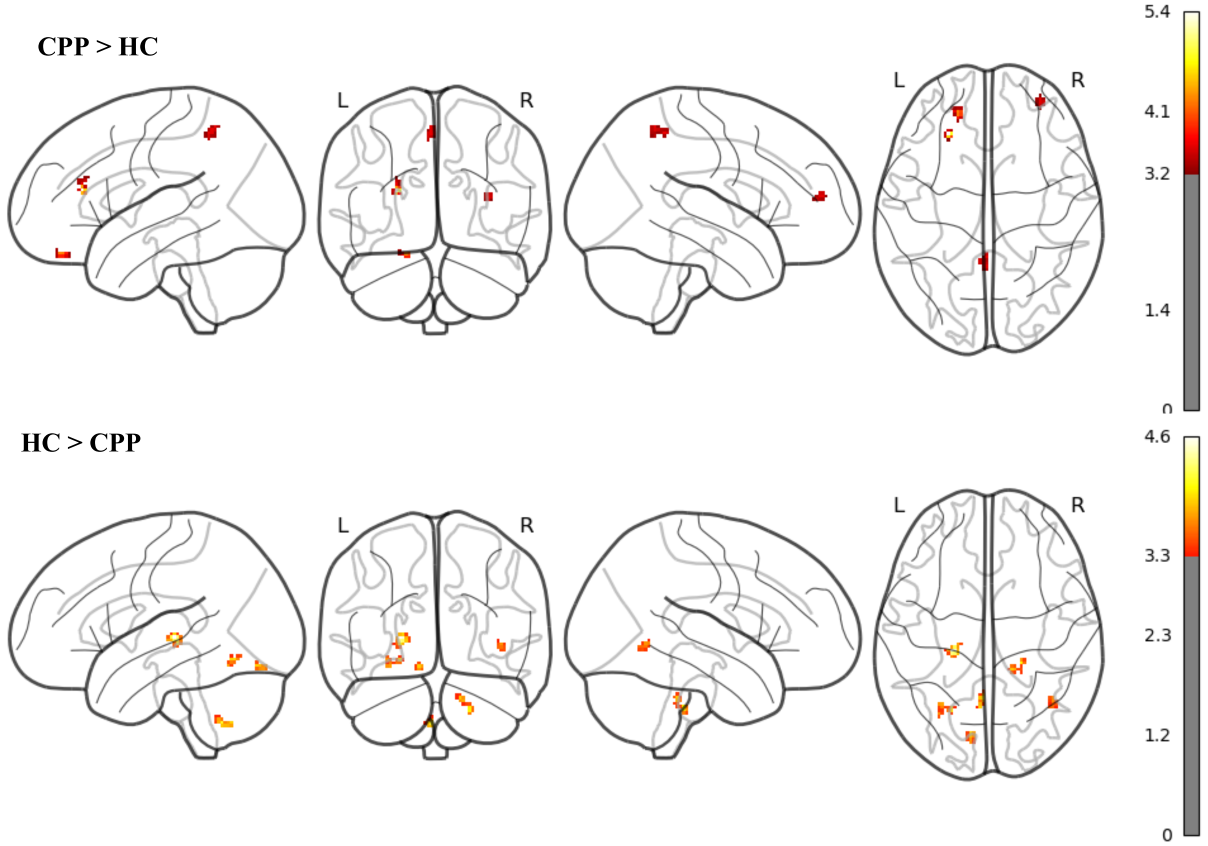

Supplementary Fig. 11. Resting-state functional connectivity of the aIC with the whole brain, presented without correction for multiple comparisons. The displayed clusters correspond to those reported in the Supplementary Table 4.

F. References

1. Rolls ET, Huang CC, Lin CP, Feng J, Joliot M. Automated anatomical labelling atlas 3. Neuroimage. 2020 Feb 1;206(116189):1–5.

2. Nieto-Castanon A, Whitfield-Gabrieli S. CONN functional connectivity toolbox: RRID SCR_009550, release 22. 2022.
